# Spatiotemporal Expression of Repulsive Guidance Molecules (RGMs) and Their Receptor Neogenin in the Mouse Brain

**DOI:** 10.1371/journal.pone.0055828

**Published:** 2013-02-14

**Authors:** Dianne M. A. van den Heuvel, Anita J. C. G. M. Hellemons, R. Jeroen Pasterkamp

**Affiliations:** Department of Neuroscience and Pharmacology, Rudolf Magnus Institute of Neuroscience, University Medical Center Utrecht, Utrecht, The Netherlands; School of Biomedical Sciences, The University of Queensland, Australia

## Abstract

Neogenin has been implicated in a variety of developmental processes such as neurogenesis, neuronal differentiation, apoptosis, migration and axon guidance. Binding of repulsive guidance molecules (RGMs) to Neogenin inhibits axon outgrowth of different neuronal populations. This effect requires Neogenin to interact with co-receptors of the uncoordinated locomotion-5 (Unc5) family to activate downstream Rho signaling. Although previous studies have reported RGM, Neogenin, and/or Unc5 expression, a systematic comparison of RGM and Neogenin expression in the developing nervous system is lacking, especially at later developmental stages. Furthermore, information on RGM and Neogenin expression at the protein level is limited. To fill this void and to gain further insight into the role of RGM-Neogenin signaling during mouse neural development, we studied the expression of RGMa, RGMb, Neogenin and Unc5A-D using *in situ* hybridization, immunohistochemistry and RGMa section binding. Expression patterns in the primary olfactory system, cortex, hippocampus, habenula, and cerebellum were studied in more detail. Characteristic cell layer-specific expression patterns were detected for RGMa, RGMb, Neogenin and Unc5A-D. Furthermore, strong expression of RGMa, RGMb and Neogenin protein was found on several major axon tracts such as the primary olfactory projections, anterior commissure and fasciculus retroflexus. These data not only hint at a role for RGM-Neogenin signaling during the development of different neuronal systems, but also suggest that Neogenin partners with different Unc5 family members in different systems. Overall, the results presented here will serve as a framework for further dissection of the role of RGM-Neogenin signaling during neural development.

## Introduction

The mammalian nervous system is composed of millions of neurons that are connected through dendritic and axonal processes. The formation of this exquisitely complex neuronal network is dependent on a precisely ordered series of developmental events including neurogenesis, neuronal differentiation and migration, neurite growth and guidance, and apoptosis. RGMs and their receptor Neogenin have been implicated in the molecular control of many of these cellular events [1]–[5]. The founding member of the RGM gene family, RGMa, was originally discovered through the biochemical characterization of a growth cone collapsing activity for chick retinal axons [6], [7]. Within the chick retinotectal system, RGMa is expressed in the retina and in an anterior-low to posterior-high gradient in the tectum. In the tectum, RGMa repels temporal retinal axons away from the posterior part of the tectum [6], [8], [9]. In addition, RGMa is required for intraretinal pathfinding of retinal axons [10]. Following the initial discovery of chick RGMa, three different RGMs were identified in mammalian species; RGMa, RGMb (also known as Dragon), and RGMc (also known as hemojuvelin (HJV), HLA-like protein involved in iron (Fe) homeostasis (HFE2), and Dragon-like muscle (DL-M)) (for review see [4]). RGMa and RGMb, but not RGMc, are expressed in the nervous system and can act as growth cone collapse factors and repulsive axon guidance cues for different populations of neurons [6,8,9,11–20–22].

Neogenin is the predominant RGM receptor in neurons. Neogenin is a member of the immunoglobulin (Ig) superfamily of cell surface proteins and a close homologue of deleted in colorectal cancer (DCC) [9], [23]. Similar to DCC, Neogenin can bind Netrin-1 and mediate Netrin-1-dependent functions [20], [24]–[27]. Interactions between RGMs and Neogenin are required for both the neuronal and non-neuronal functions of RGMa and RGMb, including their neurite growth inhibitory and axon repulsive effects [9], [20], [21], [28]–[33]. In addition, RGMs and also Neogenin interact with bone morphogenetic proteins (BMPs) and their receptors, but thus far RGM-mediated modulation of BMP signaling has not been implicated in the neurodevelopmental functions of RGMs [12], [34]–[47]. Binding of RGMa or RGMb to Neogenin on neuronal growth cones leads to activation of the Rho kinase pathway and inactivation of Ras signaling [13], [14], [48]. Interestingly, activation of RhoA by RGMs is dependent on another family of Netrin-1 receptors, Unc5s [15]. Unc5s interact with Neogenin through their extracellular domains and with leukemia-associated guanine nucleotide exchange factor (LARG), a RhoGEF, through their intracellular region. Binding of RGMa to Neogenin induces the focal adhesion kinase (FAK)-dependent tyrosine phosphorylation of LARG and as a result activation of RhoA. Neogenin can bind all four members of the Unc5 family (Unc5A-D), but only the role of Unc5B has been established at the functional level [15].

The best-characterized neuronal functions of RGMa and RGMb are in axon guidance and regeneration failure. During development, RGMs serve as repulsive axon guidance molecules in the chick retinotectal system, the mouse hippocampus and *Xenopus* forebrain [6], [8], [9], [11], [16], [20]. In addition, RGMs contribute to the control of neuronal survival [31], [49], neuron migration [28], [29], [50], [51], neuronal differentiation [8], [16], and dendritic branching and spine maturation [22]. *RGMa^−/−^* mice do not show overt defects in retinotectal mapping, as observed in chick, but display abnormalities in neural tube closure [8], [52]. Depletion of RGMa in *Xenopus* embryos also results in aberrant development of the neural tube [30]. Following injury to the adult spinal cord, RGMa and RGMb are strongly expressed around the lesion site [14], [48], [53]. Local administration of a function-blocking anti-RGMa antibody in rats significantly improves anatomical and functional spinal cord regeneration [14]. This together with their potent neurite growth inhibitory effects suggests that RGMs inhibit axon regeneration in the spinal cord. Despite these advances, the precise contribution of RGMs and Neogenin to the development of most neuronal systems remains to be explored, especially in the mouse.

Although previous studies have reported RGMa, RGMb and/or Neogenin expression in different neuronal systems and species, a systematic comparison of RGM and Neogenin expression patterns in the developing nervous system is lacking, especially at later developmental stages. Furthermore, information on RGM and Neogenin expression at the protein level is limited. In this study, we therefore used *in situ* hybridization, immunohistochemistry and RGMa section binding to perform a detailed expression analysis of RGMa, RGMb, Neogenin and Unc5A-D in a selection of neuronal systems in the mouse brain. The selected brain regions were complex multilayered structures (e.g. the olfactory system and cerebellum), connected to many other brain areas. Highly stereotypic patterns of expression were detected for RGMa, RGMb, Neogenin and Unc5A-D, including strong expression on several major axon tracts. These data support a widespread role for RGM-Neogenin signaling during neural development and suggest that Neogenin may partner with different Unc5 family members to subserve different functions in different systems. Our data, together with previous expression results, serve as a framework for further functional studies on the role of RGM-Neogenin signaling during neural development.

## Materials and Methods

### Ethics Statement

The experiments performed in this study were approved by the Experimental Animal Committee (DEC) of Utrecht University (2008.I.05.037). All animal experiments were conducted in agreement with Dutch law (Wet op de Dierproeven, 1996) and European regulations (Guideline 86/609/EEC) related to the protection of vertebrate animals used for experimental and other scientific purposes.

### Animals and Tissue Treatment

C57BL/6 mice were obtained from Charles River. Pups and (timed-pregnant) adult mice were killed by means of decapitation or cervical dislocation, respectively. The morning on which a vaginal plug was detected was considered embryonic day 0.5 (E0.5) and the day of birth, postnatal day 0 (P0). For *in situ* hybridization and RGMa section binding experiments E16.5 and P5 heads, and adult brains were directly frozen in 2-methylbutane (Merck). For immunohistochemistry, E16.5 heads were collected in phosphate-buffered saline (PBS, pH 7.4) and fixed by immersion for 3 hours (hrs) in 4% paraformaldehyde (PFA) in PBS at 4°C. P5 and adult mice were transcardially perfused with saline followed by 4% PFA. Brains were dissected and postfixed overnight at 4°C, washed in PBS, cryoprotected in 30% sucrose at 4°C and frozen in 2-methylbutane. Sections (16 µm) were cut on a cryostat, mounted on Superfrost Plus slides (Fisher Scientific), air-dried and stored desiccated at −80°C for *in situ* hybridization and at −20°C for immunohistochemistry. All mRNA and protein expression patterns and AP binding patterns were examined in at least eight embryos, pups or adult mice. Embryos or pups were derived from at least three different litters. The reported expression and binding patterns were reproducible across individual mice.

### Cell Culture and Transfection

COS-7 cells (ATTC) were maintained in high glucose Dulbecco’s modified Eagle’s medium (DMEM; Gibco, Invitrogen) supplemented with 10% (v/v) heat-inactivated fetal bovine serum (FBS; Lonza, BioWhittaker), 2 mM L-glutamine (PAA) and 1× penicillin/streptomycin (pen/strep; PAA) in a humidified atmosphere with 5% CO_2_ at 37°C. Cells were transfected with RGMa (pSectag2-RGMa-myc-his), RGMb (pSectag2-RGMb-myc-his; both were kind gifts of Silvia Arber), GFP-Neogenin (pcDNA3.1-GFP-Neogenin), GFP-DCC (a kind gift of Jean-François Cloutier), pcDNA3.1 (pcDNA3.1(-)/myc-his; Invitrogen) or pEGFP-N1 (Clontech), using polyethylenimine (PEI; Polysciences) (as described by [54]).

### AP-protein Production

For alkaline phosphatase (AP), RGMa-AP and Sema3F-AP protein production, HEK293 cells were transfected with AP-Fc (a kind gift of Roman Giger), RGMa-AP (APtag5-RGMa-AP; a kind gift of Thomas Skutella), or Sema3F-AP (a kind gift of Valerie Castellani). Transfected HEK293 cells were cultured in Opti-MEM reduced serum medium (Gibco, Invitrogen) supplemented with 3% (v/v) FBS (Lonza, BioWhittaker), 2 mM L-glutamine (PAA) and 1× pen/strep (PAA). Cell culture medium was collected after 5 days in culture, filter-sterilized and stored at 4°C. If required, culture medium containing AP-tagged proteins was concentrated using Centriprep YM-50 centrifugal filter units (Millipore).

### *In situ* Hybridization

Nonradioactive *in situ* hybridization was performed as described previously [55], with minor modification. In brief, probe sequences for *RGMa* [21], *RGMb* [21] and *Neogenin* (NM_008684.2: nt 2087–2587) were polymerase chain reaction (PCR)-amplified from cDNA, using primer sequences listed in Table S1. The probe sequences for *Unc5A* (genepaint.org: probe 1721), *Unc5B* (NM_029770.2: nt 665–1210), *Unc5C* (genepaint.org: probe 1568) and *Unc5D* [56] were generated by reverse transcription (RT)-PCR on adult mouse whole brain RNA (see Table S1). For the *tyrosine hydroxylase* (TH) probe a 1142 bp fragment of rat TH cDNA was used [57]. Digoxigenin (DIG)-labeled RNA probes were generated by a RNA polymerase reaction using 10× DIG RNA labeling mix (ENZO).

Tissue sections were postfixed with 4% PFA in PBS (pH 7.4) for 20 minutes (min) at room temperature (RT). To enhance tissue penetration and decrease aspecific background staining, sections were acetylated with 0.25% acetic anhydride in 0.1 M triethanolamine and 0.06% HCl for 10 min at RT. Sections were prehybridized for 2 hrs at RT in hybridization buffer (50% formamide, 5× Denhardt’s solution, 5× SSC, 250 µg/ml baker’s yeast tRNA and 500 µg/ml sonicated salmon sperm DNA). Hybridization was performed for 15 hrs at 68°C, using 400 ng/ml denatured DIG-labeled probe diluted in hybridization buffer. After hybridization, sections were first washed briefly in 2× SSC followed by incubation in 0.2× SCC for 2 hrs at 68°C. Sections were adjusted to RT in 0.2× SSC for 5 min. DIG-labeled RNA hybrids were detected with anti-DIG Fab fragments conjugated to AP (Boehringer) diluted 1∶2500 in Tris-buffered saline (TBS, pH 7.4) overnight at 4°C. Binding of AP-labeled antibody was visualized by incubating the sections in detection buffer (100 mM Tris-HCl, pH 9.5, 100 mM NaCl and 50 mM MgCl_2_) containing 240 µg/ml levamisole and nitroblue tetrazolium chloride/5-bromo-4-chloro-3-indolyl-phosphatase (NBT/BCIP; Roche) for 14 hrs at RT. Sections subjected to the entire *in situ* hybridization procedure, but with no probe or sense probe added, did not exhibit specific hybridization signals. Sense probe data for RGMa are shown in Fig. S1. Sense probes for other genes examined in this study displayed a similar amount of background staining. The specificity of the *in situ* hybridization procedure was also inferred from the clearly distinct gene expression patterns observed. Staining was visualized using a Zeiss Axioskop 2 microscope.

### Immunocytochemistry

COS-7 cells were fixed with 4% PFA for 15 min at RT, washed in PBS (pH 7.4) and permeabilized and blocked in normal blocking buffer (PBS, 4% bovine serum albumin (BSA) and 0.1% Triton) for 1 hr at RT. COS-7 cells were incubated with goat anti-RGMa antibody (AF2458; R&D systems) 1∶200, sheep anti-RGMb antibody (AF3597; R&D systems) 1∶50 or goat anti-Neogenin antibody (AF1079; R&D systems) 1∶50 in normal blocking buffer for 2 hrs at RT. Cells were washed in PBS and incubated with the appropriate Alexa Fluor-labeled secondary antibodies (Invitrogen) 1∶500 at RT. After 1 hr, cells were washed in PBS and counterstained with with 4′, 6′-diamidino-2-phenylindole (DAPI; Invitrogen).

### Immunohistochemistry

Immunohistochemistry was performed as described previously [58], [59]. In brief, sections were washed in PBS (pH 7.4) and incubated in normal blocking buffer (PBS, 4% BSA and 0.1% Triton) for 1 hr at RT and incubated with goat anti-RGMa antibody (AF2458; R&D systems) 1∶200, sheep anti-RGMb antibody (AF3597; R&D systems) 1∶200 or goat anti-Neogenin antibody (AF1079; R&D systems) 1∶200 overnight in normal blocking buffer at 4°C. The specificity of the RGMa and Neogenin antibodies has been confirmed previously using immunocytochemical, immunohistochemical and/or Western blot methods on transfected cells and endogenous tissues [38], [60]–[63]. As a control, sections were incubated with immunoglobulin isotype controls matching the RGM or Neogenin antibodies (AB-108-C, 5-001-A; R&D systems). For costainings with glial fibrillary protein (GFAP), rabbit anti-GFAP (Z0334; DAKO) 1∶6000 was used. The next day, sections were washed in PBS and incubated with the appropriate Alexa Fluor-labeled secondary antibodies (Invitrogen) 1∶500 for 1 hr at RT. Sections were washed in PBS, counterstained with fluorescent Nissl stain (NeuroTrace, Invitrogen) 1∶500 for 15 min at RT, washed in PBS and embedded in Mowiol (Sigma-Aldrich). Staining was visualized using a Zeiss Axioskop 2 microscope.

### Section Binding

Sections were fixed by immersion in −20°C methanol for 6 min and rehydrated in TBS+ (TBS, pH 7.4, 4 mM MgCl_2_ and 4 mM CaCl_2_). Section were incubated in blocking buffer (TBS+ and 10% FBS (Lonza, BioWhittaker) for 1 hr at RT and incubated with 1.5 nM AP-Fc or AP-tagged protein-containing medium for 2 hrs at RT. After washing in TBS+, sections were incubated with fixation solution (20 mM HEPES, pH 7, 60% (v/v) acetone and 3.7% formaldehyde) for 2 min. After washing in TBS+, endogenous phosphatase activity was heat-inactivated by incubation at 65°C for 1 h. Section were equilibrated in detection buffer (100 mM Tris-HCl, pH 9.5, 100 mM NaCl and 5 mM MgCl_2_) and bound AP-protein was visualized by incubation in detection buffer containing levamisole and NBT/BCIP (Roche). The specificity of RGMa-AP protein binding was determined by competition with excess RGMa protein. Furthermore, differential binding patterns were observed following RGMa-AP and Sema3F-AP section binding and no staining was observed for AP-Fc alone.

## Results

To provide an overview of the expression of RGMa, RGMb and Neogenin during mouse neural development, we used a combination of *in situ* hybridization, immunohistochemistry and RGMa section binding. *In situ* hybridization not only revealed gene expression in specific structures and cell layers, but also aided in the identification of the cellular source of RGM or Neogenin protein expression as revealed by immunohistochemistry and allowed for comparison to previously reported gene expression patterns. Given the role of Unc5s as obligate RGM co-receptors [15], expression of *Unc5A-D* was also studied. Unfortunately, no suitable antibodies are available to perform immunohistochemistry for all Unc5s, therefore *in situ* hybridization was used [64].

Not much is known about the expression of RGM and Neogenin protein in the developing brain, therefore immunohistochemistry was used to reveal RGM and Neogenin protein expression in glial cells, neurons and their processes. Finally, given the ability of RGMs to bind cell surface receptors other than Neogenin [36], RGMa-AP (alkaline phosphatase) section binding was used to examine whether RGMa binding sites in the brain correspond to regions of Neogenin expression. Three different timepoints were selected for these studies: E16.5, as an early timepoint during which developmental processes such as neurogenesis, cell migration and axon guidance occur; postnatal day (P)5, characterized by late developmental processes such as synapse formation, pruning and apoptosis; and adulthood, to explore a possible role for RGM-Neogenin signaling in the plasticity of mature neuronal networks. The specificity of the observed expression patterns could be discerned from the various controls that were included (e.g. sense controls, use of isotype immunoglobulin controls, omission of primary antibody, section binding with AP only) and from the clearly distinct expression patterns. The subsequent sections discuss expression profiles in a selection of neuronal systems displaying the most prominent RGM and Neogenin expression patterns in the mouse. The expression patterns reported here are largely in line with those reported in previous studies and apparent discrepancies are discussed if data from equivalent stages and species is available.

### Primary Olfactory System

**Figure 1:**
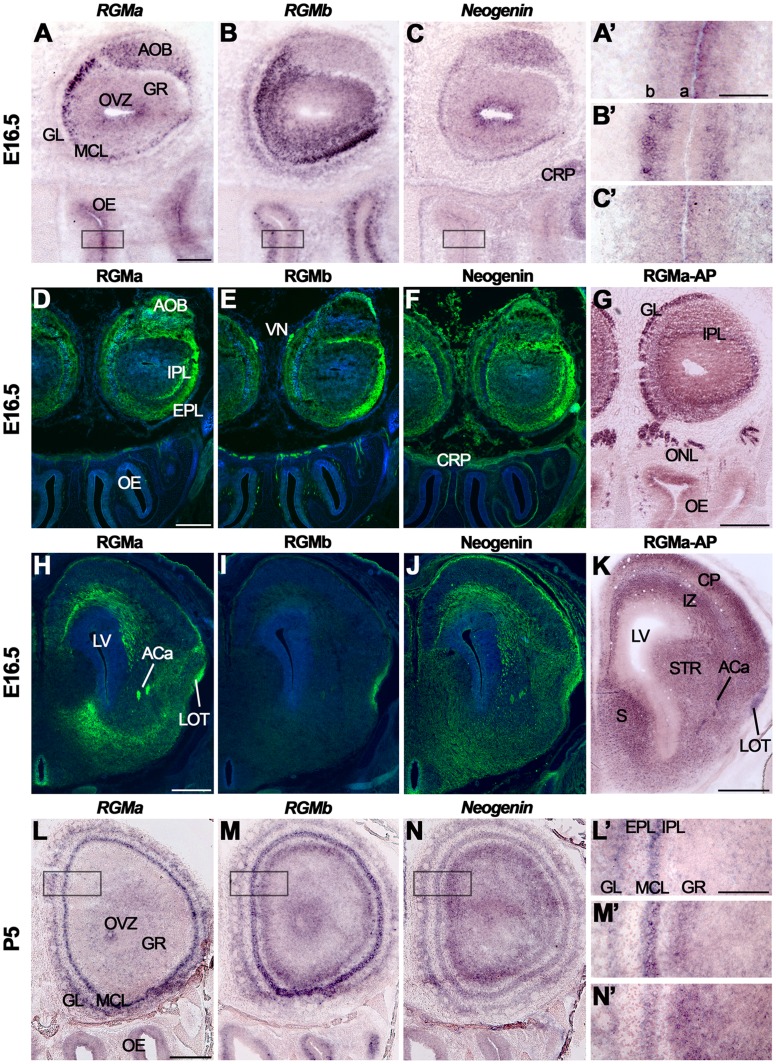
RGM and Neogenin expression in the mouse olfactory system. *In situ* hybridization on coronal mouse brain sections at E16.5 (A–C′) and P5 (L–N′). Panels A′–C′ and L′–N′ show higher magnifications of boxed areas in A–C and L–N, respectively. Immunohistochemistry (D–F, H–J) and RGMa-AP section binding (G, K) on E16.5 coronal mouse brain sections. Sections in D–F and H–J are counterstained in blue with fluorescent Nissl. (A–C′) *In situ* hybridization shows differential expression patterns of *RGMa*, *RGMb* and *Neogenin* in the olfactory bulb and olfactory epithelium (OE). In line with this, immunohistochemistry reveals that axons of olfactory sensory neurons in the OE stain strongly for RGMb and weakly for RGMa and Neogenin. Furthermore, RGMa, RGMb and Neogenin are expressed on olfactory bulb axon projections such as the lateral olfactory tract (LOT). a, apical; ACa, anterior commissure pars anterior; AOB, accessory olfactory bulb; b, basal; CP, cortical plate; CRP, cribriform plate; EPL, external plexiform layer; GL, glomerular layer; GR, granule cell layer; IPL, internal plexiform layer; IZ, intermediate zone; LV, lateral ventricle; MCL, mitral cell layer; ONL, olfactory nerve layer; OVZ, olfactory ventricular zone; S, septum; STR, striatum; VN, vomeronasal nerve. Scale bar A–C 200 µm, A′–C′ 100 µm, D–F 300 µm, G 500 µm, H–J 400 µm, K 500 µm, L–N 400 µm and L′–N′ 200 µm.

Olfactory sensory neurons (OSNs) in the olfactory epithelium (OE) express receptors for the detection of odorants and synapse their axons on mitral cell dendrites in select regions of the olfactory bulb, termed glomeruli [65]. Mitral cells then relay the olfactory information to higher brain structures [66]. *In situ* hybridization showed complementary expression patterns for *RGMa* and *RGMb* in the OE and olfactory bulb at E16.5 (Fig. 1A, A′, B, B′, Table S2). *RGMa* was most strongly expressed in the apical part of the OE and *RGMb* in its basal part. Weak expression of *Neogenin* was detected in the OE with strongest signals in the apical cell layer (Fig. 1C, C′). In the olfactory bulb, expression of *RGMa* was found in a dorsomedial subset of mitral cells, while *RGMb* was most strongly expressed in ventrolateral mitral cells (Fig. 1A, B). Weak *Neogenin* expression was present in the mitral cell layer (MCL) and the cribriform plate (Fig. 1C). In addition, *RGMa* and *Neogenin* were detected in the olfactory ventricular zone and the accessory olfactory bulb (Fig. 1A, C). *RGMb* was expressed at low levels in the accessory olfactory bulb but strongly in the granule cell and glomerular layers (GR and GL, respectively) (Fig. 1B). These expression patterns are largely unchanged at E18.5 [19], [67].

To examine the expression of RGMs and Neogenin on the axonal projections of OSNs and mitral cells, immunohistochemistry was used. The specificity of the RGMa and Neogenin antibodies has been confirmed previously using immunocytochemical, immunohistochemical and/or Western blot methods on transfected cells and endogenous tissues [38], [60]–[63]. Here, the specificity of the anti-RGM and anti-Neogenin antibodies was further tested by immunocytochemistry (Fig. S2) and by the inclusion of immunoglobulin isotype controls matching the RGM or Neogenin antibodies. Antibodies directed against RGMa detected RGMa but not RGMb, and vice versa. Anti-Neogenin antibodies specifically recognized Neogenin but not its close family member DCC (Fig. S2). The use of immunoglobulin isotype controls or the omission of primary antibodies resulted in the absence of specific signals. Immunohistochemistry at E16.5 revealed strong expression of RGMb and weak staining for RGMa and Neogenin on OSN axons in the OE and olfactory bulb glomeruli (Fig. 1D–F). In general, we found that the RGM and Neogenin antibodies more strongly labeled axonal projections as compared to cell bodies. Interestingly, previous work reports Neogenin protein expression in the basal part of the E14.5 OE, apparently contrasting the *in situ* data at E16.5 [68] (Fig. 1C′). It is possible that this difference is caused by a spatiotemporal change in Neogenin expression or by the use of different antibodies in the present study and that of Fitzgerald *et al*. [68]. The antibody used here recognizes the N-terminal part of Neogenin, while in the other study antibodies are used against the Neogenin C-terminal region. In relation to this it is interesting to note that Neogenin can be cleaved resulting in the release of the extracellular domain [61]. This may also explain differences in expression patterns. In the olfactory bulb, RGMa, RGMb and Neogenin were observed in mitral cell axons in the internal and external plexiform layers (IPL and EPL) (Fig. 1D–F). Neogenin expression was also observed in the cribriform plate, in line with expression of *Neogenin* transcripts in this structure (Fig. 1C, F). RGMa-AP bound to the IPL, EPL and GL, resembling Neogenin expression (Fig. 1F, G). RGMb was also detected in the vomeronasal nerve (Fig. 1E). Mitral cells organize their axons in the lateral olfactory tract (LOT) en route to more caudal targets in the central nervous system. RGMa, RGMb and Neogenin were expressed by axons in the LOT (Fig. 1H–J). In addition, axons in the anterior commissure (pars anterior) (ACa), which connects olfactory structures to the anterior piriform cortex, strongly stained for RGMa and Neogenin (Fig. 1H, J). RGMb was weakly expressed in the ACa (Fig. 1I). RGMa-AP strongly bound to the LOT and the ACa (Fig. 1K).

At P5 and adult stages, *in situ* hybridization revealed strong expression of *RGMa*, *RGMb* and *Neogenin* in periglomerular cells in the GL and in the MCL (Fig. 1L–N, L′–N′, Table S2). In the GR, *RGMb* and *Neogenin* were most strongly expressed in granule cells located adjacent to the IPL (Fig. 1M, M′, N, N′). Expression patterns in the OE were as observed at E16.5 (Fig. 1A–C′, L–N). Immunohistochemistry revealed expression of RGMa, RGMb and Neogenin on OSN axons in the GL. The GR expressed RGMa and Neogenin and the vomeronasal nerve was prominently stained for RGMb. Mitral cell axons in the olfactory bulb and LOT expressed RGMa, RGMb and Neogenin. The ACa expressed RGMa and Neogenin (data not shown).

**Figure 2:**
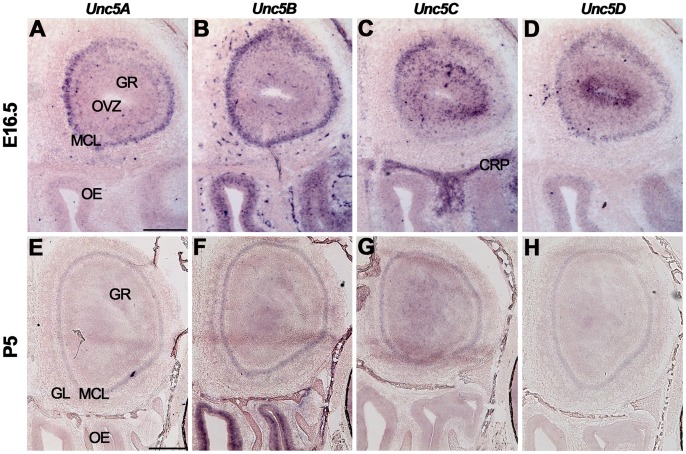
Unc5 expression in the olfactory system. *In situ* hybridization on coronal mouse brain sections at E16.5 (A–D) and P5 (E–H). All *Unc5s* are differentially expressed in the olfactory bulb but the olfactory epithelium (OE) only expresses *Unc5B* and *Unc5D*. CRP, cribriform plate; GL, glomerular layer; GR, granule cell layer; MCL, mitral cell layer; OVZ, olfactory ventricular zone. Scale bar A–D 300 µm and E–H 400 µm.

The expression of *Unc5A-D* was studied using *in situ* hybridization (Fig. 2, Table S2). At E16.5, expression of all four *Unc5* family members was observed in the MCL of the olfactory bulb (Fig. 2A–D). Interestingly, *Unc5C* was only expressed by a small subset of dorsal mitral cells (Fig. 2C). *Unc5B* staining was observed in the OE and in blood vessels, in line with its proposed role in angiogenesis (Fig. 2B) [69]. *Unc5C* was detected in the GR and the cribriform plate (Fig. 2C). *Unc5D* was strongly expressed in the olfactory ventricular zone and weakly in the OE and GR (Fig. 2D). At P5 and adult stages, expression levels of *Unc5A-D* were reduced as compared to E16.5, but signals remained in the GL, MCL and GR (Fig. 2E–H, Table S2).

### Cortex

The adult mammalian cerebral cortex consists of six layers comprised of morphologically and functionally distinct neurons. These layers are formed between E11 and E18, as cortical neurons undergo radial migration from the ventricular progenitor zone to their final position in the cortex [70]. The cortex is the origin of several major axon tracts in the forebrain, including the corticothalamic and corticospinal tracts. Cortical expression of *RGMa*, *RGMb* and *Neogenin* has been reported and RGMa can inhibit the outgrowth of cortical axons *in vitro* through Neogenin [13], [15], [18], [19], [22], [67], [71], [72]. However, the precise role of RGMs and Neogenin during different stages of cortical development and maturation *in vivo* is still incompletely understood.

**Figure 3:**
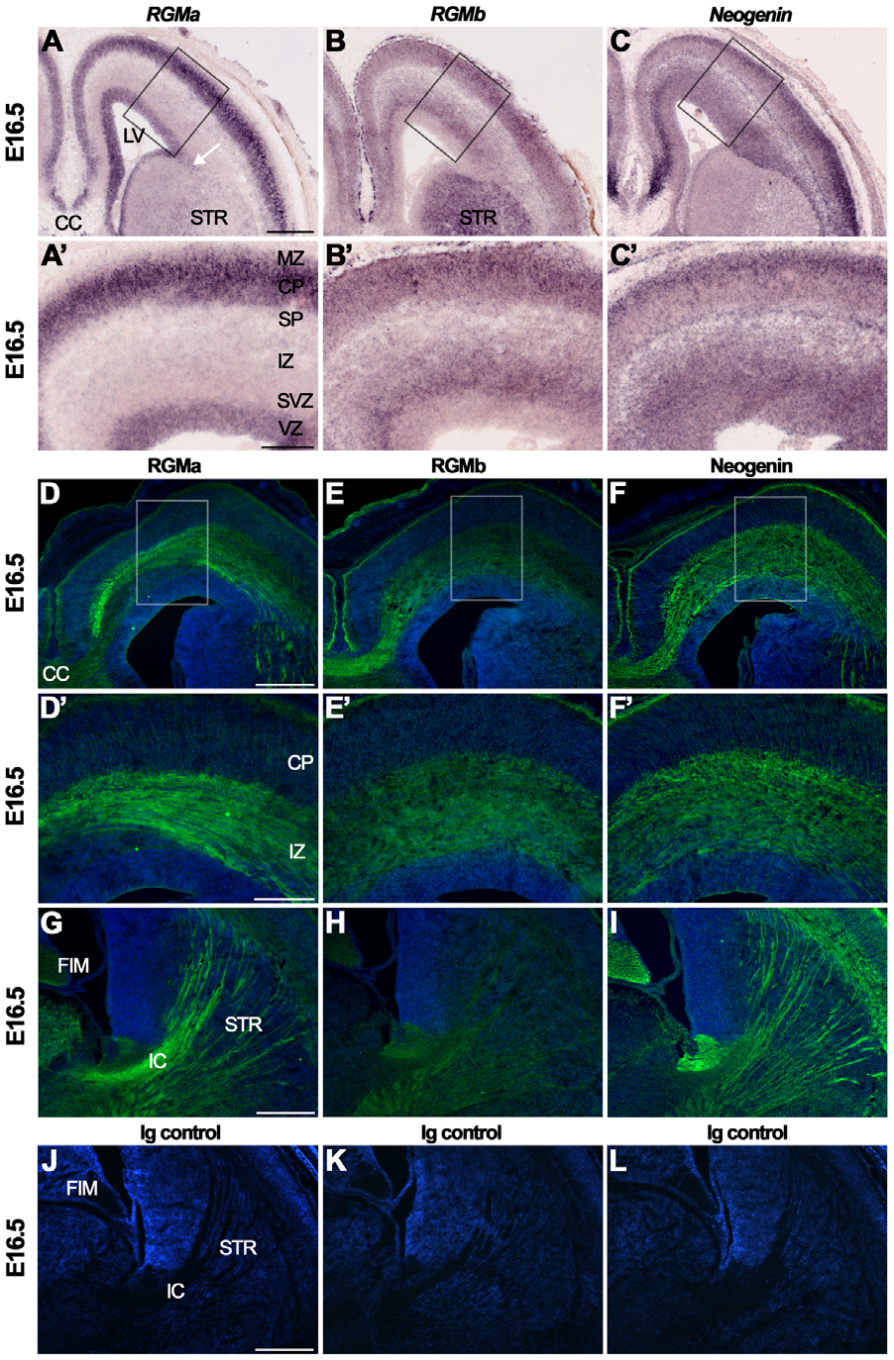
RGMa, RGMb and Neogenin display partially complementary patterns of expression in the developing cortex and on cortical projections. *In situ* hybridization (A–C′) and immunohistochemistry (D–L) on coronal mouse brain sections at E16.5. Panels A′–C′ and D′–F′ show higher magnifications of the boxed areas in A–C and D–F, respectively. Sections in D–L are counterstained in blue with fluorescent Nissl. (A–C′) *In situ* hybridization reveals strong expression of *RGMa* and *Neogenin*, and moderate expression of *RGMb*, in the embryonic mouse cortex. Arrow in A indicates neurons of the lateral migratory stream. (D–I) RGMa and Neogenin protein are strongly expressed in the cortex and on various cortical axon projections. Strong staining for RGMb (E), and Neogenin (F), is detected in the corpus callosum (CC). The internal capsule (IC) stains strongly for RGMa (G). (J–L) Immunostaining with isotype-matched control antibodies did show significant staining. CP, cortical plate; FIM, fimbria; IZ, intermediate zone; LV, lateral ventricle; MZ, marginal zone; SP, subplate; STR, striatum; SVZ, subventricular zone; VZ, ventricular zone. Scale bar A–C 400 µm, A′–C′ 200 µm, D–F 300 µm, D′–F′ 150 µm, G-I 300 µm and J-M 300 µm.

At E16.5, expression of *RGMa* was restricted to the cortical plate (CP) and the ventricular zone (VZ) (Fig. 3A, A′, Table S3). *RGMb* expression was strongest in the pia, the upper part of the CP, and the subventricular zone (SVZ) (Fig. 3B, B′) [19]. *Neogenin* was present throughout the developing cortex with prominent expression in the subplate (SP) and the upper CP (Fig. 3C, C′). This pattern is similar to that reported at E14.5 and E18.5 [18], [19]. Immunohistochemistry at E16.5 revealed distinct and complementary expression patterns for RGMa and RGMb on cortical projections. Strong expression of RGMa was found on axons in the intermediate zone (IZ) and the internal capsule (IC). Weak RGMa labeling was present on axons traversing the striatum and on axons of the corpus callosum (CC) (Fig. 3D, D′, G). Expression of RGMb was weak on axons in the IZ, IC and striatum, but strong at the level of the CC (Fig. 3E, E′, H). Neogenin was found on axons in the IZ, CC, and on axons traversing the striatum (Fig. 3F, F′, I). Interestingly, Neogenin-positive axons occupied the outer part of the IC, whereas RGMa-positive axons traversed its central part (Fig. 3G, I). Since RGMa, but not Neogenin, is strongly expressed in the dorsal thalamus, the IC labeling for RGMa is likely to represent thalamocortical axon projections. It should be noted that expression of RGMs and Neogenin on axon projections was more prominent as compared to the staining of cell bodies in the CP that gave rise to these projections. Interestingly, assessment of the cortical expression of Neogenin at E14.5 using an antibody directed against the C-terminal part of Neogenin revealed a different, more widespread pattern of expression including strong labeling of the ventricular zone [68], [71], [72]. It will therefore be interesting to determine whether this difference arises from the cleavage of Neogenin [61]. In line with previous observations, RGMa and Neogenin were also expressed on cells with the appearance of radial glia cells in the CP and in cells in the VZ (Fig. 3D′, F′) [50], [72]. Strong binding of RGMa-AP was detected in the upper CP, in the striatum, and on axon bundles in the IZ and the IC (Fig. 1K, S3A, B). These observations are in line with the expression of Neogenin detected by *in situ* hybridization and immunohistochemistry in these brain areas (Fig. 3C, C′, F, F′, I). Sections incubated with isotype immunoglobulin controls did not show specific expression (Fig. 3J–L).

**Figure 4:**
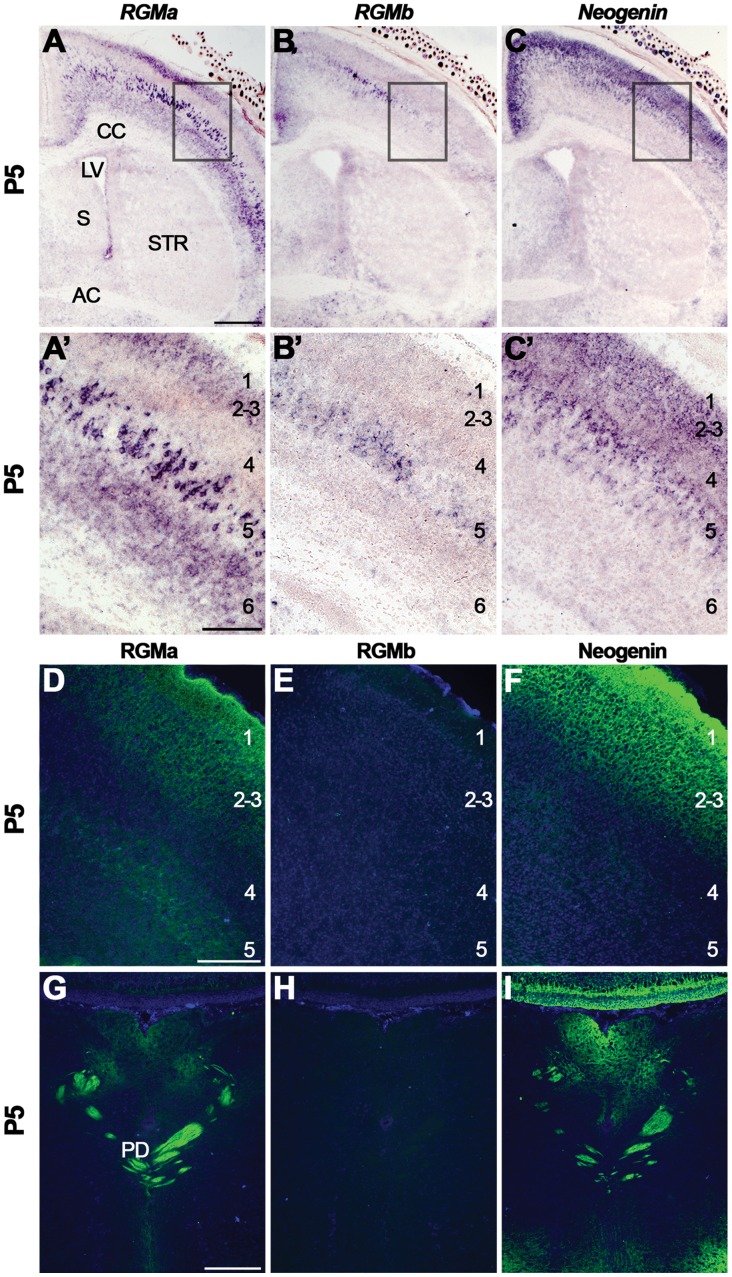
Postnatal expression of RGMa, RGMb and Neogenin in the cortex and corticospinal tract. *In situ* hybridization (A–C′) and immunohistochemistry (D–I) on coronal mouse brain sections at P5. Panels A′–C′ show higher magnifications of boxed areas in A–C. Sections in D-I are counterstained in blue with fluorescent Nissl. (A–C′) *In situ* hybridization detects strong expression of *RGMa* in cortical layers 1–3, 5 and 6. *RGMb* is mainly expressed in layer 5 in a medial to lateral gradient and *Neogenin* is expressed in layers 1–5. (D–F) RGMa protein is expressed in layers 1–3 and 5. Very weak staining is detected for RGMb and Neogenin is strongly expressed in cortical layers 1–3. (G–I) High levels of RGMa and Neogenin are detected in the corticospinal tract. AC, anterior commissure; CC, corpus callosum; LV, lateral ventricle; PD, pyramidal decussation; S, septum; STR, striatum. Scale bar A–C 600 µm, A′–C′ 200 µm, D–F 200 µm and G–I 250 µm.

At P5, *RGMa* was expressed in layers 1–3, 5 and 6 of the cortex and expression of *RGMb* was confined to neurons of layer 5 displaying a medial high to lateral low gradient (Fig. 4A, A′, B, B′) [18]. *Neogenin* was expressed in layers 1–5 (Fig. 4C, C′, Table S3). Immunohistochemistry detected staining for RGMa in layers 1–3 and 5 (Fig. 4D). RGMb was virtually absent from the P5 cortex and Neogenin staining was most prominent in layers 1–3 (Fig. 4E, F). RGMa, RGMb and Neogenin were expressed on axons in the CC and the IC (data not shown). Strong immunostaining for RGMa and Neogenin was also detected in the corticospinal tract (CST), which is formed by cortical efferents from layer 5 cortical neurons (Fig. 4G–I). In the adult, expression of *RGMa* was detected in layers 5–6 and the VZ while *RGMb* and *Neogenin* were expressed throughout the cortex (Table S3). However, no specific signals for RGMa, RGMb or Neogenin were detected in the adult cortex by immunohistochemistry (data not shown).

**Figure 5:**
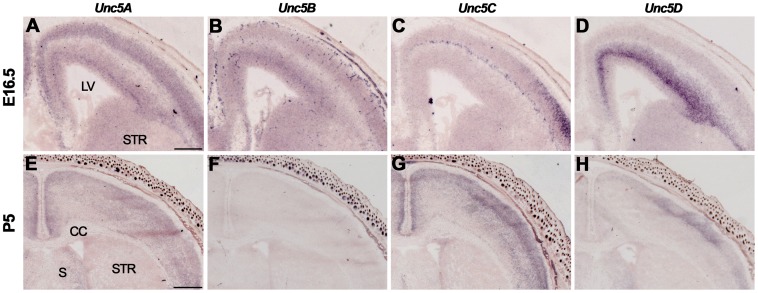
Unc5 expression in the cortex. *In situ* hybridization on coronal mouse brain sections at E16.5 (A–D) and P5 (E–H). (A–D) *Un5A-C* are expressed in the cortical plate (CP), and *Unc5A* and *Unc5C* in the subplate (SP). All *Unc5*s are expressed in the subventricular zone (SVZ) and ventricular zone (VZ). (E–H) At P5, *Unc5A*, *Unc5C* and *Unc5D* are expressed in the cortex. CC, corpus callosum; LV, lateral ventricle; S, septum; STR, striatum. Scale bar A–D 300 µm and E-H 600 µm.

At E16.5, *Unc5A-C* were expressed in the CP, *Unc5A* and *Unc5C* in the SP, and *Unc5A-D* in the SVZ and VZ (Fig. 5A–C, Table S3). Expression of *Unc5D* was especially strong in the SVZ (Fig. 5D). At P5 and in adulthood, *Unc5A* was expressed throughout the cortex, *Unc5D* in layers 1–4 and no signals for *Unc5B* were detected [73] (Fig. 5E, F, H, Table S3). *Unc5C* was expressed throughout the cortex at P5 but was restricted to layer 5 in the adult (Fig. 5G).

### Hippocampus

The hippocampus is a multilayered structure with an essential role in learning and memory. It receives afferent projections from different regions in the brain. Axons from entorhinal cortex (CEn) neurons project via the perforant pathway to the molecular layer (ML) of the dentate gyrus (DG) and via the alvear pathway to the stratum lacunosum moleculare (SLM). Axons from the septum project to the stratum oriens (SO) and stratum radiatum (SR). Within the hippocampus, mossy fibers from DG granule cells synapse on cornu ammonis (CA)3 pyramidal neurons, while Schaffer collaterals from CA3 neurons project to the CA1 region [74].

**Figure 6:**
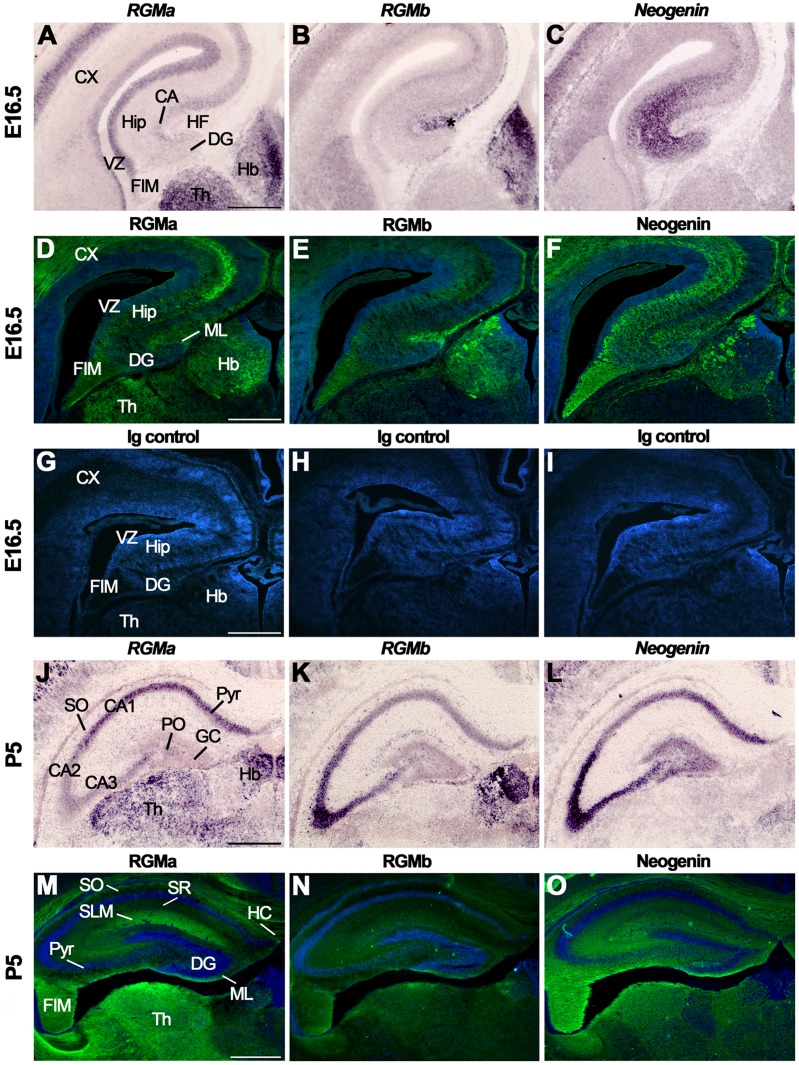
Subregion-specific expression of RGMs and Neogenin in the hippocampus. *In situ* hybridization (A–C, J–L) and immunohistochemistry (D–I, M–O) on coronal mouse brain sections at E16.5 (A–I) and P5 (J–O). Sections in D–I and M–O are counterstained in blue with fluorescent Nissl. (A–F) RGMa mRNA and protein are expressed in the ventricular zone (VZ), dentate gyrus (DG) and cornu ammonis (CA) region. Strong expression of RGMb mRNA and protein is detected in the pial surface lining the hippocampal fissure (HF). Neogenin transcripts and protein are widely expressed in the developing hippocampus (Hip). (G–I) Immunostaining with isotype matched controls. (J–L) *In situ* hybridization at P5 shows strong but differential expression patterns of *RGMa*, *RGMb* and *Neogenin* in the CA pyramidal cell layers (Pyr). In addition, strong expression of *Neogenin* is detected in the granular layer (GC) of the DG. (M–O) Immunohistochemistry reveals expression of RGMa and weak expression of RGMb in the stratum lacunosum moleculare (SLM) and fimbria (FIM). Neogenin strongly labels different hippocampal layers. CX, cortex; Hb, habenula; HC, hippocampal commissure; PO, polymorph layer; SO, stratum oriens; SR, stratum radiatum; Th, thalamus. Scale bar A–C: 400 µm, D–F: 300 µm, G–I: 300 µm, J–L: 500 µm and M–O: 400 µm.

At E16.5, *RGMa* was strongly expressed in the hippocampal VZ and specifically labeled neurons in the DG and CA layers (Fig. 6A, Table S4). Although weak to moderate *RGMb* expression was detected throughout the hippocampal formation, *RGMb* was prominently expressed in the pial layer lining the hippocampal fissure (Fig. 6B). Strong expression of *Neogenin* was observed in the DG, CA region and dentate migration stream. Lower signals were present in the SVZ and VZ (Fig. 6C) (for E18.5 patterns see [67]). Previous work has shown that pial RGMb expression directs the migration of Neogenin-positive granule cells in the dentate migratory stream [28], [67]. Sections incubated with isotype immunoglobulin controls did not show specific expression (Fig. 6G–I). At E16.5, *RGMa*, *RGMb* and *Neogenin* were expressed in the CEn and the septum (Table S4, S5). Immunohistochemistry detected RGMa in the hippocampal VZ, CA region, fimbria (FIM) and in the inner ML of the DG at E16.5 (Fig. 6D). *In vitro* studies suggest that RGMa expression in the inner ML of the DG functions to restrict Neogenin-positive CEn axons to the outer ML [11]. Weak RGMb expression was detected in the CA region, DG and FIM. However, in line with the *in situ* hybridization data, strong staining was detected in the pia (Fig. 6B, E). Neogenin was present in the outer ML of the DG, CA region, VZ and FIM (Fig. 6F). In line with this pattern of expression, RGMa-AP bound to the DG, CA region and FIM (Fig. S3A, B).

Hippocampal expression of *RGMs* and *Neogenin* persisted at P5 (Fig. 6J–L, Table S4). In line with previous work, strongest expression of *RGMa* was detected in CA1 neurons, while *RGMb* expression was most prominent in the CA2 and CA3 region (Fig. 6J, K) [11], [18]. *Neogenin* was detected in CA2 and CA3 neurons and in a medial high to lateral low expression gradient in the CA1 region (Fig. 6L). Granule cells in the DG displayed weak expression of *RGMa* and *RGMb,* and strong *Neogenin* expression (Fig. 6J–L). *RGMa*, *RGMb* and *Neogenin* were expressed in the polymorph layer (PO) of the DG (Fig. 6J–L). Immunohistochemistry at P5 revealed expression of RGMa in the SO, SLM, FIM, ML and hippocampal commissure (Fig. 6M). Weak expression of RGMb was detected in the SLM, FIM and the outer ML of the DG (Fig. 6N). Neogenin was detected throughout the hippocampus, including in the FIM, hippocampal commissure, SLM, SO, SR and in the ML, granular layer and PO of the DG (Fig. 6O). Adult hippocampal expression of *RGMa*, *RGMb* and *Neogenin* resembled that observed at P5. However, *Neogenin* expression levels were decreased in the adult and *RGMa*, *RGMb* and *Neogenin* were absent from the SR and ML (Table S4). Furthermore, it should be noted that another study failed to detect *RGMa* expression in the adult hippocampus [19]. Immunohistochemistry in the adult hippocampus revealed weak expression of RGMa and RGMb in the SLM. Neogenin was prominently expressed in the PO and SR. Expression of RGMa, RGMb and Neogenin in the adult FIM was reduced as compared to P5 (data not shown).

**Figure 7:**
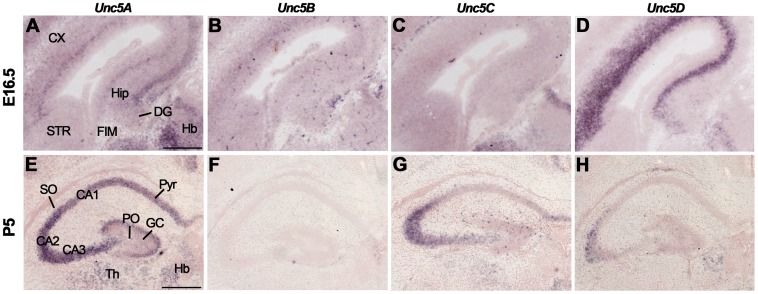
Unc5 expression in the hippocampus. *In situ* hybridization on coronal mouse brain sections at E16.5 (A–D) and P5 (E–H). (A–D) *Unc5A-D* are expressed in the E16.5 hippocampus (Hip) and dentate gyrus (DG). (E–H) At P5, *Unc5A* and *Unc5C* are expressed in cornu ammonis (CA) 1–3 and *Unc5D* expression is restricted to CA3. CX, cortex; FIM, fimbria; GC, granular layer; Hb, habenula; HC, hippocampal commissure; PO, polymorph layer; Pyr, pyramidal cell layers; SO, stratum oriens; SR, stratum radiatum; STR, striatum; Th, thalamus. Scale bar A–C: 400 µm and D–F: 300 µm.

Although Unc5A expression has been reported in hippocampal mossy fibers [75], the expression of Unc5 family members during hippocampal development is largely unknown. We detected *Unc5A-D* expression in different regions of the E16.5 hippocampus with highest *Unc5A-D* expression in the CA region and DG (Fig. 7A–D, Table S4). Of the four Unc5 family members, *Unc5A* displayed the most prominent expression in the developing hippocampus. At P5 and in the adult, *Unc5A-D* signals were detected in CA1-CA3 and the DG, though staining for Unc5B was not detected in the P5 hippocampus (Fig. 7E–H, Table S4).

### Habenula

The habenula is part of the epithalamus and is subdivided into a medial (MHb) and lateral part (LHb). The MHb receives major inputs from the septum and the LHb receives afferents from the basal ganglia. The fasciculus retroflexus (FR) is the main output bundle of the Hb and carries LHb and MHb axons to the midbrain [76]. The expression and role of RGMs and Neogenin during the development of the habenula and its projections are unknown.

**Figure 8:**
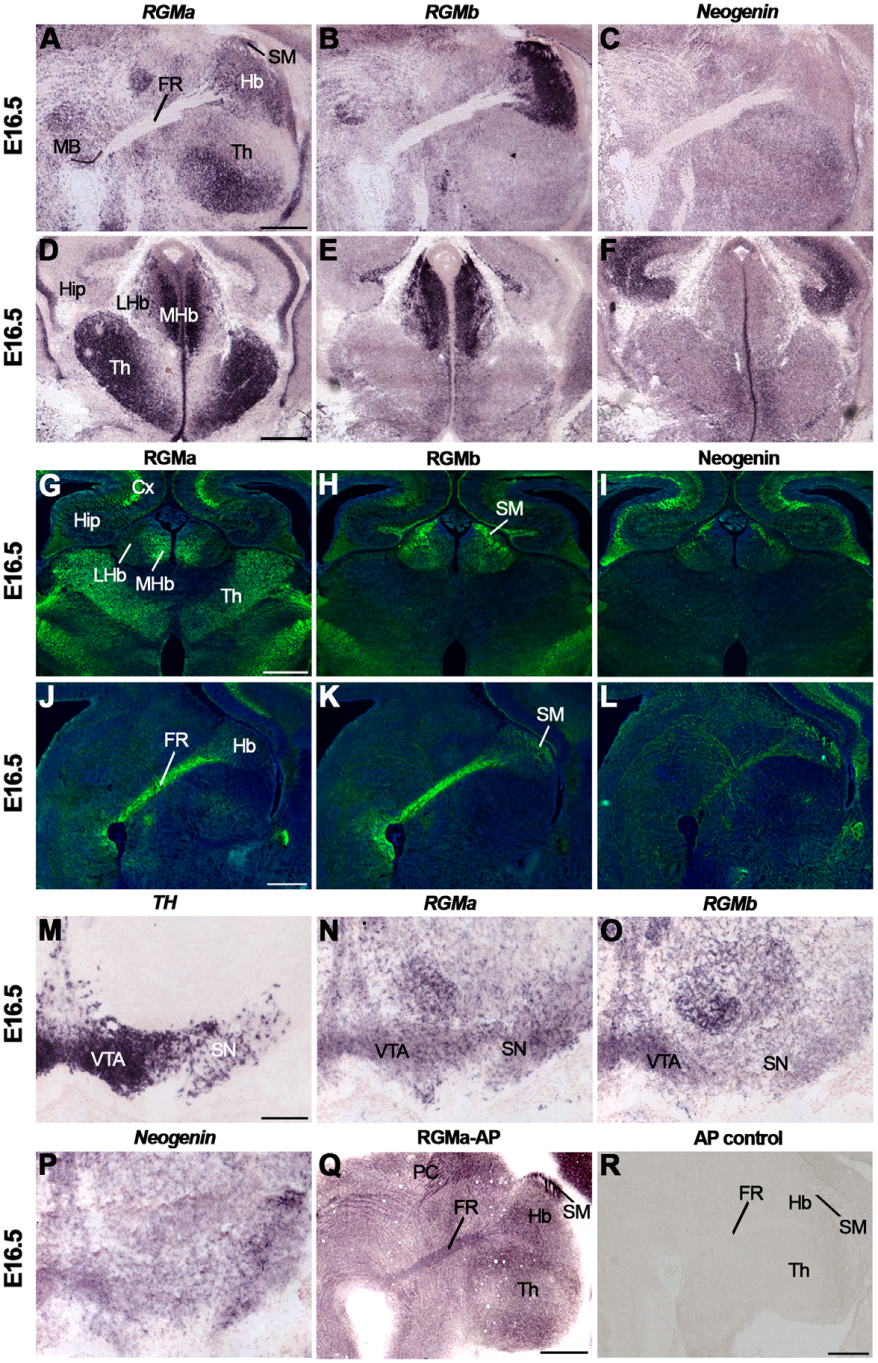
Differential expression of RGMs and Neogenin in the habenula and its efferent and afferent projections. *In situ* hybridization (A–F, M–P), immunohistochemistry (G–L) and RGMa-AP (Q) and AP (R) section binding on coronal (A–I, M–P) and sagital (J–L, Q–R) mouse brain sections at E16.5. Sections G–L are counterstained in blue with fluorescent Nissl. (A–L) *In situ* hybridization and immunostaining reveal strong expression of RGMa in the medial habenula (MHb) and strong expression of RGMb in the MHb and lateral habenula (LHb). Weak Neogenin expression is detected in the LHb and MHb. In line with this, strong RGMa and RGMb immunostaining is detected on the fasciculus retroflexus (FR), the major output bundle of the Hb. (M–P) *In situ* hybridization for *tyrosine hydroxylase* (*TH*) stains dopaminergic neurons in the substantia nigra (SN) and ventral tegmental area (VTA). *RGMa* and *Neogenin* expression is detected in the SN and VTA, while *RGMb* is predominantly expressed in the VTA. (Q) RGMa-AP section binding shows strong staining of the stria medullaris (SM) and weak staining of the FR. (R) Section binding with AP control. CX, cortex; Hip, hippocampus; MB, midbrain; PC, posterior commissure; Th thalamus. Scale bars A–L: 400 µm, M–P: 200 µm and Q–R: 400 µm.

At E16.5, *in situ* hybridization revealed strong *RGMa* expression in the MHb and lower signals in the LHb. Strong *RGMb* and weak *Neogenin* expression was detected in the MHb and in the medial part of the LHb (Fig. 8A–F). In addition, strong expression for *RGMa* was observed in the thalamus and for *RGMb* in the striatum (Fig. 8D, Table S5). Several of the synaptic targets of LHb and MHb axons in the FR expressed *RGMa*, *RGMb* and *Neogenin*, including the interpeduncular nucleus and the mesodiencephalic dopamine system (Table S5). Interestingly, the mesodiencephalic dopamine system not only receives habenular inputs but also projects axons to the LHb [77], [78]. At E16.5, *RGMa* was expressed in the ventral tegmental area (VTA) and substantia nigra (SN), which were identified by *tyrosine hydroxylase* (TH) labeling (Fig. 8M, N). Strong expression of *RGMb* was detected in the VTA and *Neogenin* was present in a subset of neurons in the VTA and SN (Fig. 8O, P). Immunohistochemistry revealed that RGMa expression was confined to the MHb while RGMb and Neogenin were expressed in the MHb and LHb (Fig. 8G–I). Furthermore, strong staining of the FR for RGMa and RGMb and only weak expression of Neogenin was detected (Fig. 8J–L). RGMb and Neogenin were also expressed in the stria medularis (SM) (Fig. 8H, I). The SM contains afferent projections to the Hb from different brain areas several of which display high levels of *Neogenin* and *RGMb* (e.g. the septal nuclei and lateral hypothalamic region) (Table S5). RGMa-AP section binding detected moderate to weak binding to the FR and strong binding to the SM. Sections incubated with AP control did not exhibit specific staining (Fig. 8Q, R).

**Figure 9:**
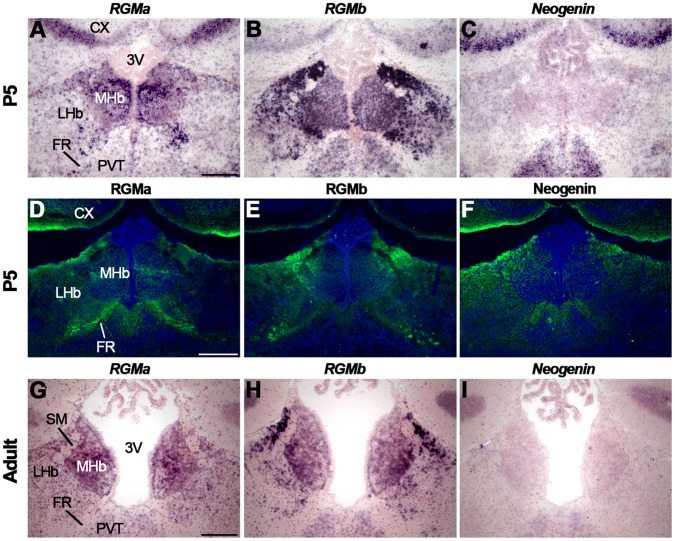
Postnatal RGM and Neogenin expression in the habenula and fasciculus retroflexus. *In situ* hybridization (A–C, G–I) and immunohistochemistry (D–F) on coronal mouse brain sections at P5 (A–F) and in the adult (G–I). Sections D–F are counterstained in blue with fluorescent Nissl. (A–C, G–I) At P5 and in the adult, *in situ* hybridization reveals strong expression of *RGMa* and *RGMb* in the medial habenula (MHb). The lateral habenula (LHb) strongly expresses *RGMb*, while only weak expression of Neogenin is detected in Hb. (D–F) Immunohistochemistry detects strong expression of RGMa and RGMb and weak expression of Neogenin in the fasciculus retroflexus (FR). 3V, third ventricle; CX, cortex; SM, stria medullaris; PVT, paraventricular thalamic nucleus. Scale bars A–I: 200 µm.

At P5 and in the adult, strong *RGMa* expression was observed in the MHb and *RGMb* expression was most prominent in the LHb and the lateral aspect of the MHb (Fig. 9A, B, G, H, Table S5) (see [18] for an equivalent pattern at P7). Only weak expression of *Neogenin* was detected (Fig. 9C, I). Expression of *RGMs* and *Neogenin* was also detected in the paraventricular thalamic nucleus, just ventral to the Hb (Fig. 9A–C, G–I). Immunohistochemistry revealed expression of RGMa and RGMb, and weak expression of Neogenin in the FR at P5 and in the adult (Fig. 9D–F, data not shown).

**Figure 10:**
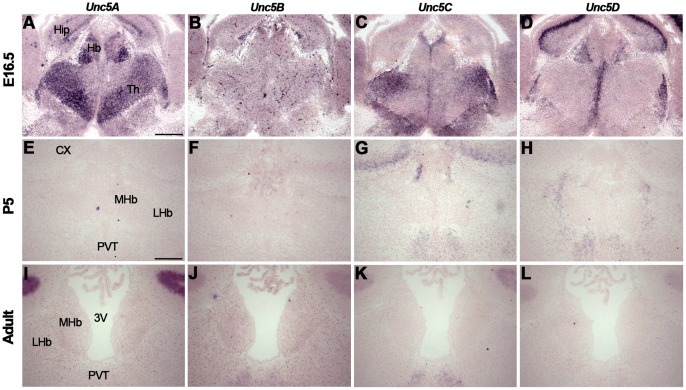
Unc5 expression in the habenula. *In situ* hybridization on coronal mouse brain sections at E16.5 (A–D), P5 (E–H) and adult (I–L). (A–D) At E16.5 *in* situ hybridization shows expression of *Unc5A-D* in the habenula (Hb). *Unc5B* expression labels blood vessels but not habenular neurons. (E–H) At P5, *Unc5A* is weakly expressed in the Hb and *Unc5D* expression is restricted to the lateral habenula (LHb). (I–L) In the adult Hb, weak *Unc5B* expression is detected. 3V, third ventricle; CX, cortex; MHb, medial habenula; PVT paraventricular thalamic nucleus. Scale bar A–D: 300 µm and E–L: 200 µm.

*In situ* hybridization at E16.5 revealed strong expression of *Unc5A* in the LHb and MHb (Fig. 10A, Table S5). Weak expression of *Unc5B* was detected in the Hb, particularly staining blood vessels, and expression of *Un5C* and *Unc5D* was confined to the LHb (Fig. 10B–D). At P5, *Unc5A* was weakly expressed in the MHb and LHb and expression of *Unc5D* was restricted to the LHb (Fig. 10E, H, Table S5). Expression of *Unc5B* and *Unc5C* was not detected in the Hb (Fig. 10F, G). In adult, almost no *Unc5* staining was observed in the Hb, except for very weak *Unc5B* labeling (Fig. 10I–L, Table S5).

### Cerebellum

The cerebellum is located in the hindbrain and is associated with motor coordination and controlled movement. The adult cerebellum consists of three different layers: the molecular layer (ML), the Purkinje cell layer (PCL) and the granular cell layer (GCL). Interestingly, the neurons that occupy these layers derive from two different progenitor zones. The cerebellar VZ gives rise to Purkinje cells (PCs), Bergmann glia (BG) and interneurons. Granule cells precursors (GCPs) are generated in the upper rhombic lip lining the fourth ventricle and migrate tangentially along the cerebellar surface to form the external granular layer (EGL). During the first two postnal weeks, cerebellar granule cells (CGCs) migrate from the EGL radially along BG in the ML to the internal granular cell layer (IGL) [79].

**Figure 11:**
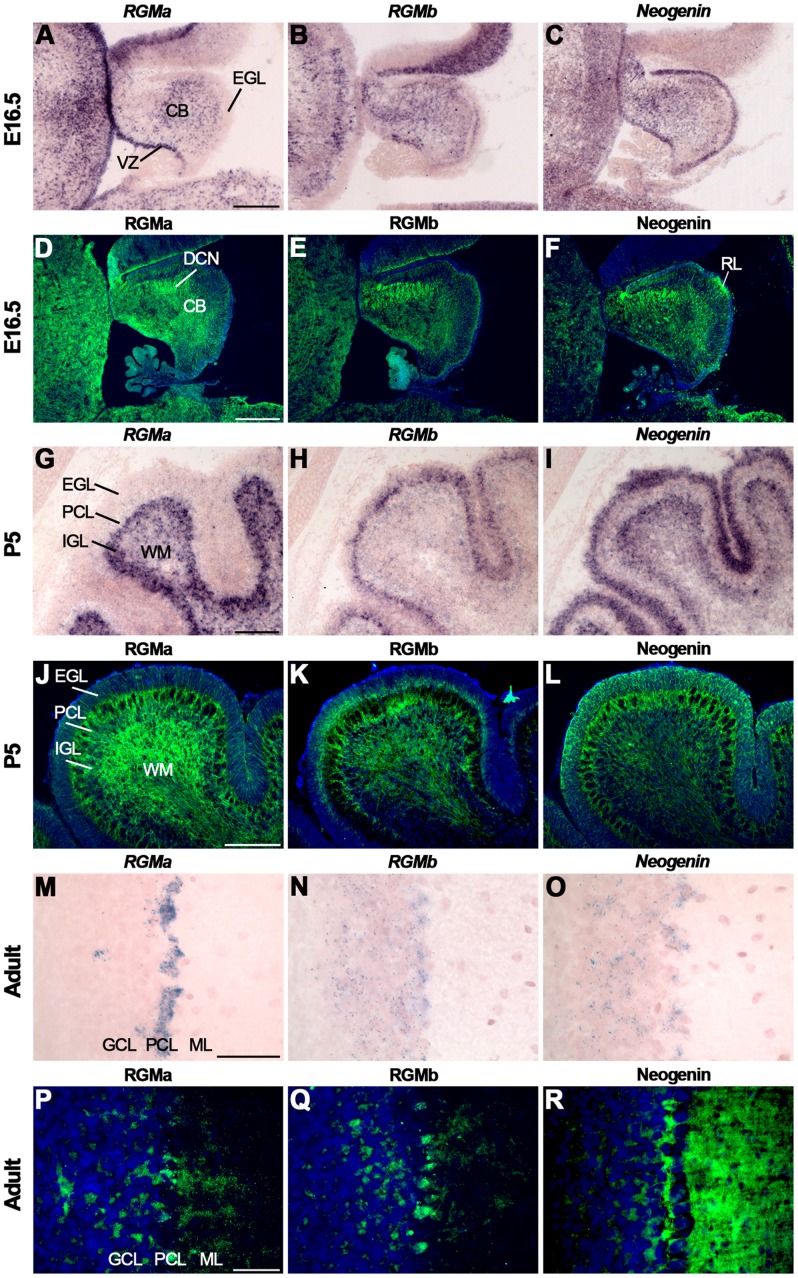
Differential expression of RGMs and Neogenin in the cerebellum. *In situ* hybridization (A–C, G–I, M–O) and immunohistochemistry (D–F, J–L, P–R) on coronal mouse brain sections at E16.5 (A–F), P5 (G–L) and in the adult (M–R). (A–I, M–O) *In situ* hybridization and immunohistochemistry reveals strong and broad expression of RGMa, RGMb and Neogenin in the cerebellum (CB). (J–L) Immunostaining shows expression of RGMa and Neogenin in all cerebellar layers at P5. RGMb is expressed in the internal granular layer (IGL), Purkinje cell layer (PCL) and external granular layer (EGL). (P–R) In the adult, RGMa, RGMb and Neogenin protein are expressed in Purkinje cells (PCs) and axons in the granular cell layer (GCL), PCL and molecular layer (ML). Neogenin strongly labels PC dendrites in the ML. DCN, deep cerebellar nuclei; WM, white matter; VZ, ventricular zone. Scale bar A–C: 300 µm, D–F: 250 µm, G–I: 150 µm, J–L: 150 µm, M–O: 100 µm and P–R: 50 µm.

*In situ* hybridization at E16.5 revealed *RGMa* expression in the VZ of the cerebellum but not in the EGL (Fig. 11A, Table S6). *RGMb* was expressed in a subset of cells in the inner part of the EGL and *Neogenin* staining was detected in the VZ and throughout the EGL (Fig. 11B, C) [18], [19], [23]. This *RGM* expression profile is in line with previously reported expression patterns at E14.5 [18]. Interestingly, however, at E18.5 cerebellar *RGMb* signals are already much more restricted as compared to the expression observed at E16.5 (Fig. 11B) [19]. Furthermore, expression of *Neogenin* in the EGL has been reported previously at E13 and is in line with RGMa-AP binding patterns (Fig. S3C), but could not be detected at E18.5 [23], [67]. *RGMa*, *RGMb* and *Neogenin* were expressed in the PCL and deep cerebellar nuclei (DCN) (Fig. 11A–C). In line with this expression, immunostaining revealed expression of RGMa, RGMb and Neogenin in the DCN (Fig. 11D–F). RGMa was also expressed in cellular processes traversing the EGL (Fig. 11D). In line with the *in situ* hybridization data, RGMb was confined to the inner part of the EGL (Fig. 11E). Neogenin expression was detected throughout the EGL and in a patch of GCPs in the EGL where the presumptive rhombic lip is located (Fig. 11F) [23].

**Figure 12:**
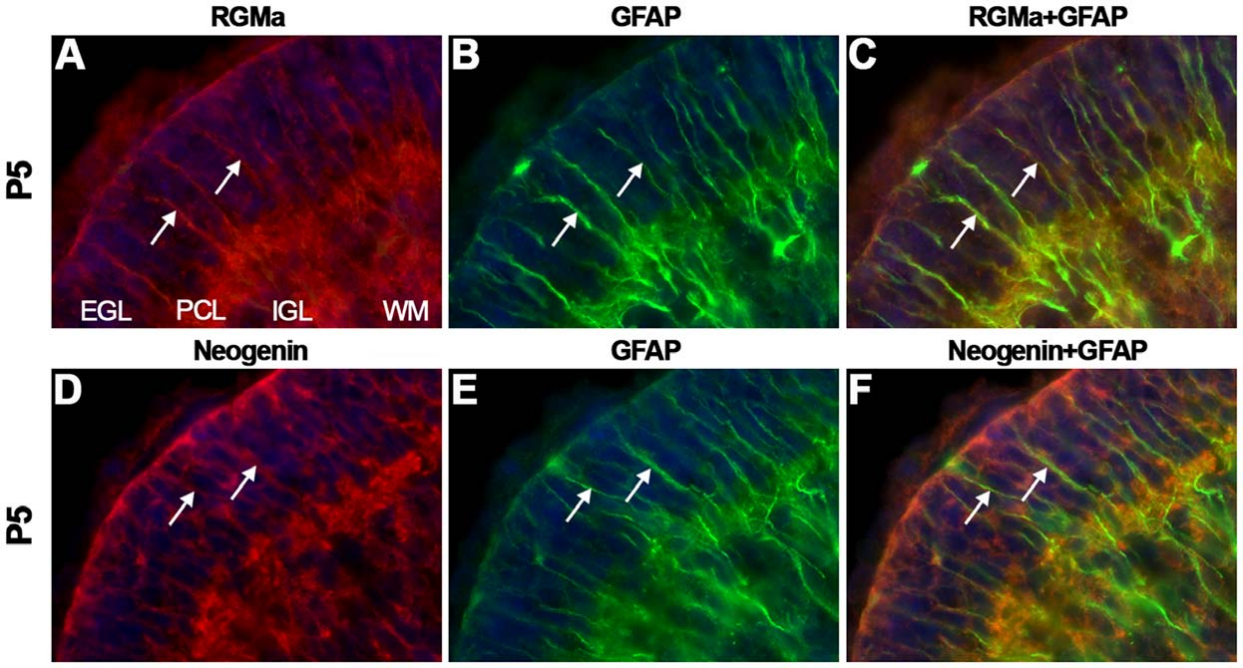
RGMa and Neogenin are expressed on Bergmann glia. Immunohistochemistry for RGMa (A, C), glial fibrillary acidic protein (GFAP) (B–C, E–F) and Neogenin (D, F) on coronal mouse brain sections at P5 visualized by confocal microscopy. Sections are counterstained in blue with fluorescent Nissl. (A–F) RGMa and Neogenin immunostaining (in red) colocalizes with GFAP-positive staining (in green) on Bergmann glial fibers (arrows). Granule cells in the external granular layer (EGL) also express Neogenin. IGL, internal granular layer; PCL, Purkinje cell layer; WM, white matter.

*At P5, RGMa* was confined to the IGL (Fig. 11G, Table S6). *RGMb* was detected in the inner EGL, PCL and at lower levels in the IGL (Fig. 11H). *Neogenin* was strongly expressed in the outer part of the EGL, in the PCL and IGL (Fig. 11I). Immunohistochemistry showed staining for RGMa, RGMb and Neogenin in the EGL (Fig. 11J–L). Furthermore, RGMa, RGMb and Neogenin were expressed in the PCL and IGL including the white matter tracts in this structure. However, since no *RGMa* expression was detected in the PCL (Fig. 11G) this labeling may represent expression on migrating cerebellar granule neurons or BG. To determine whether RGMs and Neogenin are expressed on the radial processes of BG, co-immunostaining for glial fibrillary acidic protein (GFAP), a BG marker, and RGMa or Neogenin was performed. Both RGMa and Neogenin expression colocalized with the GFAP-positive BG fibers in the EGL (Fig. 12). In addition, cerebellar granule neurons along BG processes expressed Neogenin.

In the adult, *in situ* hybridization revealed expression of *RGMa*, *RGMb* and *Neogenin* in the PCL and GCL (Fig. 11M–O, Table S6). It should be noted that other work does not detect RGMb in the adult cerebellum [19]. Immunohistochemistry revealed staining for RGMa, RGMb and Neogenin in the GCL, PCs and the ML. Staining in the ML may represent the dendritic processes of PCs (Fig. 11P–R).

**Figure 13:**
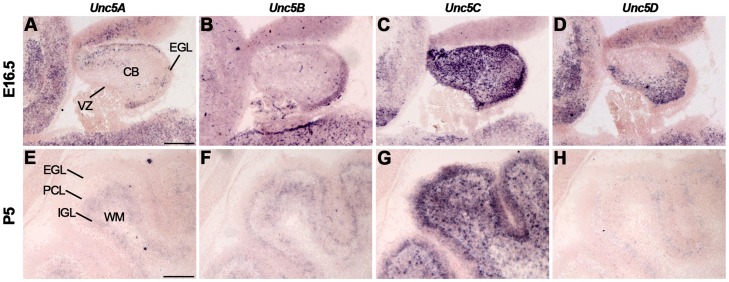
Dynamic expression of Unc5 in the developing cerebellum. *In situ* hybridization on coronal mouse brain sections at E16.5 (A–D) and P5 (E–H). (A–D) Expression of *Unc5A-D* is detected in the cerebellum (CB), although expression of *Unc5C* is most prominent. *Unc5A-C* are expressed in the external granular layer (EGL). (E–H) At P5, *Unc5B* and *Unc5C* are expressed in the EGL, Purkinje cell layer (PCL) and internal granular layer (IGL). Expression of *Unc5B* in the EGL is restricted to the inner cell layers. Expression of *Unc5A* is only detected in the IGL and expression of *Unc5D* is restricted to the PCL. VZ, ventricular zone; WM, white matter. Scale bar A–D: 300 µm and E–H: 150 µm.

Expression of Unc5A-D in the cerebellum has been reported in embryonic and postnatal stages and in the adult [64], [80]–[86]. *Unc5C* knockout mice display severe defects in cerebellar development, including disturbed GCP migration and ectopically located PCs [80], [83], [84], [87]. In line with these observations, *Unc5A-C* but not *Unc5D* were expressed in the EGL at E16.5 (Fig. 13A–D, Table S6). At P5, *Unc5A* was restricted to the IGL, while *Unc5B* and *Unc5C* were detected in the EGL, PCL and IGL (Fig. 13E–G). Weak expression of *Unc5D* was only detected in the PCL (Fig. 13H). In adult *Un5A-C* were expressed in the PCL and IGL, while expression of *Unc5D* is only detected in the PCL (Table S6).

## Discussion

Since their original identification in 2002, RGMs have been implicated in several different aspects of neural development. A large part of this work has focused on the important roles of RGMa and its receptor Neogenin during axon pathfinding in the chick retinotectal system [6], [8]–[10], [52]. However, experiments in *Xenopus* embryos and on cultured rodent neuronal tissues have also highlighted more widespread roles for RGM-Neogenin signaling in axon guidance [11]–[17], [20]. In addition, it has become clear that RGMs are pleiotropic and can regulate processes such as neurogenesis, differentiation, migration, and apoptosis [8], [28], [29], [31], [32], [49]–[51], [88]. Despite this progress, the precise role of RGMs and Neogenin in the development of many neuronal systems remains unknown, especially in the mouse. To begin to provide further insight into the possible roles of RGM-Neogenin signaling during mouse brain development, we performed a comparative analysis of the expression of RGMa, RGMb and Neogenin transcript and protein at different developmental stages. Unc5s were included in this analysis as they are obligate co-receptors for the axon repulsive effects of RGMs [15].

### Neurogenesis, Differentiation and Migration

Previous work has shown expression of RGMs and Neogenin in the proliferative zones of different brain structures [11], [18], [28], [50], [51], [67], [72], [89]. Our data confirm and extend these findings and reveal expression of Neogenin, RGMs and Unc5s in the progenitor regions of the olfactory epithelium, olfactory bulb, cortex, hippocampus and cerebellum. For example, strong Neogenin expression was detected in the upper part of the cerebellar EGL, which contains progenitors for CGCs [90], [91], and in the VZ of the olfactory bulb (Fig. 1C). Of the two RGMs expressed in the brain, RGMa was especially prominent in progenitor regions. On the other hand, expression of RGMb often appeared to mark regions containing differentiating cells immediately adjacent to the progenitor zones. For example, RGMb expression was detected in the SVZ of the cortex while RGMa was expressed in the adjacent VZ (Fig. 3A′, B′) [18], [19]. Together these observations support widespread roles for RGMa and RGMb in neurogenesis and neuronal differentiation.

The moment that neuroblasts start to differentiate often coincides with their migration towards their target areas. The first indication that Neogenin regulates cell migration comes from the analysis of morpholino-induced Neogenin knockdown in zebrafish, which show defects in neural tube formation and somitogenesis [92]. It is clear, however, that RGM-Neogenin signaling also plays a crucial role in cell migration at later developmental stages. This is nicely illustrated by the ability of RGMb, expressed along the hippocampal fissure, to guide Neogenin-positive DG granule cells though the DG migratory stream towards the hippocampus [28]. Such a role for RGMs and Neogenin is likely to be more general. For example, we, and others, report Neogenin expression on radially migrating neurons in the cortex and young interneurons tangentially migrating from the ganglionic eminence (GE) [51], [93]. Furthermore, Neogenin is expressed in migrating olfactory interneuron precursors in the rostral migratory stream [50], [94]. The role of RGM-Neogenin signaling in these populations of migrating neurons is largely unknown. Repulsive signaling induced by RGMs may guide cortical interneurons during their migration from the GE to the cortex. A recent study shows that overexpression of Neogenin in the GE leads to a failure of interneurons to migrate out of the GE [93]. This migrational defect may be caused by an increased response of Neogenin-overexpressing cells to the attractive effects of Netrin-1, which is expressed in the GE [93]. Alternatively, however, increased Neogenin levels may enhance the responsiveness of interneurons to repulsive cues around the GE. An interesting candidate is RGMb, which is expressed in the striatum around the time of interneuron migration (Fig. 3B). Overexpression of Neogenin in interneurons may render these cells more sensitive to the repulsive effects of RGMb thereby confining them to the GE.

Another region where Neogenin and RGMs may regulate cell migration is the cerebellum. GCPs are generated in the rhombic lip and then migrate to the surface of the cerebellum to form the EGL. From this outer part of the EGL CGCs change their mode of migration from tangential to radial and migrate along radial glial projections towards the IGL. Strong *Neogenin* expression is detected in radially migrating CGCs in the outer EGL (Fig. 11I). In contrast, *Neogenin* is absent from CGCs in the inner EGL, where CGCs switch from tangential to radial migration. Interestingly, strong expression of RGMb is detected in the inner EGL complementary to Neogenin expression in the outer EGL (Fig. 11H). It is therefore tempting to speculate that RGMb-Neogenin signaling is involved in the switch from tangential to radial migration. Of the Unc5 family members, Unc5C has been shown to have an important role in cerebellar development. *Unc5C* is strongly expressed in the developing cerebellum [86] and mutations in the *Unc5C* gene result in severe defects in cerebellar development, including a reduction of cerebellar size, abnormal cerebellar foliation and ectopic localization of PCs and CGCs [80], [83], [84], [87]. Analysis of *the Unc5C* mutant mice reveals that Unc5C regulates migration of GCPs along the caudo-rostral and dorso-ventral axes. GCPs expressing mutant Unc5C invade the superior colliculus and brain stem [80], [83]. A possible explanation for these migrational defects is the inability of migrating GCPs to respond to repellent guidance cues in the environment. In line with this hypothesis, RGMs are expressed in the superior colliculus and in the VZ of the cerebellum [18], [19], [52] and are therefore in the appropriate location to repel Neogenin- and Unc5C-expressing migrating GCPs and restrict their migration to ‘future cerebellar brain areas’. Further work is needed to examine whether or how interactions between RGMs, Neogenin and Unc5s regulate cerebellar development.

### Axon Guidance and Axon Tract Development

RGMa- and RGMb-Neogenin signaling mediates neurite outgrowth inhibition of cultured chick retinal ganglion and mouse cortical, entorhinal cortical, cerebellar granule and dorsal root ganglia (DRG) neurons [6], [9], [11], [12], [14], [15], [17]. However, our understanding of the role of these neurite outgrowth inhibitory effects in regulating axon guidance events *in vivo* is far from complete. Our study together with previous work shows that RGMs and Neogenin are abundantly expressed throughout the developing mouse brain [18], [19], [67], indicating a potential role in regulating axon guidance events in different brain areas. Neogenin expression was detected in many different axonal tracts in the brain, including the LOT, cortical efferents, the ACa and CST, and axonal tracts in the hippocampus (fimbria) and cerebellum. Interestingly, many axon tracts also showed staining for RGMa and RGMb. The LOT, ACa, IC, CST, FR and axonal tracts in the hippocampus and cerebellum were strongly stained for RGMa. The LOT and FR also expressed RGMb and strong expression of RGMb was detected in OSN axons and the CC.

Axonal expression of RGMs may aid in organizing axon bundles into sub-bundles or in creating exclusion zones for axons. For example, clear differential expression of RGMs and Neogenin was found on axon bundles in the IC. In the IC, expression of RGMa is predominant in the core, while Neogenin expression is detected in axonal tracts in the outer regions of the IC (Fig. 3G, I). This suggests that axonally expressed RGMa may instruct Neogenin-positive axons to grow in the outer parts of the IC. In addition, axonal RGM expression may mediate the adhesion of axons into tight bundles. Evidence for a possible role for RGMb as an adhesive cue for DRG axons comes from a coculture assay of Neogenin- and RGMb-positive DRG neurons and HEK-293 cells transiently expressing RGMb. In this assay, DRG neurites make contacts with RGMb-expressing HEK293 cells [95]. Furthermore, RGMb enhances the adhesion of dissociated cultured DRG neurons to HEK293 cells transiently expressing RGMb. RGMa can also exert adhesive effects through Neogenin, for example during neural tube closure [30], [52], [88], [92]. Further experiments are needed to confirm the role of these potential homophilic and heterophilic RGM interactions in axon tract development *in vivo*.

### Neuronal RGM Receptor Complexes

The inhibitory effects of RGMs on neurite outgrowth depend on a multimeric receptor complex containing Neogenin and Unc5s. Although Unc5B has been shown to be required for these effects at the functional level, Neogenin can bind all Unc5 family members [15]. This suggests that Neogenin may interact with different Unc5s in different systems, cellular processes and/or at developmental stages. Our comparative analysis of *Unc5A-D* co-receptors during brain development revealed prominent and differential expression patterns at all developmental stages studied. At E16.5 multiple brain regions were identified that displayed expression of all *Unc5s*, including the olfactory bulb, hippocampus and hypothalamus (see Tables S2, S3, S4, S5, S6). However, even within these regions expression of *Unc5s* was often highly specific and confined to specific substructures. For example in the VZ of the E16.5 olfactory bulb, prominent expression of *Unc5D* and Neogenin was detected, while expression of *Unc5A-C* was absent (Fig. 1C, F, 2A–D, Table S2). This invites the speculation that a Neogenin/Unc5D receptor complex may play a role in neuronal cell proliferation and neurogenesis in the olfactory bulb. In the habenula, we detected very specific expression of *Unc5A* in the MHb, while the LHb expressed *Unc5A*, *Unc5C* and *Unc5D* at E16.5 (Fig. 10A–D, Table S5). This indicates differential roles for Unc5s in the development of MHb and LHb neurons. Although binding of all Unc5 molecules to Neogenin has been shown, it is currently not known whether binding of different Unc5s results in different functional outcomes. Future work will focus on examining whether different Unc5 proteins serve as functional co-receptors for RGM receptors in different brain regions and during different developmental processes.

### Conclusion

This study presents a comparative analysis of the expression of RGMa, RGMb, Neogenin and Unc5A-D in the mouse brain at three key developmental stages (E16.5, P5 and adult). The observed expression patterns support a widespread, and largely unexplored, role for RGMa/b and their Neogenin/Unc5 receptor complex in neuron proliferation, migration and axon guidance in the mouse brain. Interestingly, RGMs may function both as exogenous cues that are detected by cells or neurites or as axon-derived factors involved in axon tract development. Analysis of Unc5 expression patterns suggests that the composition of the RGM receptor complex, i.e. which Unc5 family member it contains, may differ between different brain regions and/or cellular processes. In all, these data serve as a valuable framework for the further dissection of the role of RGMs during mouse neural development.

## Supporting Information

Figure S1 **No specific staining for sense probes.** *In situ* hybridization on coronal mouse brain sections at E16.5 (A–B), P5 (C–D) and in adulthood (E–F) using *RGMa* sense probes. No specific *in situ* hybridization signals were detected at any of the timepoints or in any of the brain regions examined. Sections hybridized with sense probes for *RGMb*, *Neogenin*, and *Unc5A-D* displayed similar levels of background labeling (not shown). 3V, third ventricle; CP, cortical plate; CX, cortex; GCL, granular cell layer; Hb, habenula; Hip, hippocampus; IZ, intermediate zone; LHb, lateral habenula; MHb, medial habenula; ML, molecular layer; PVT, paraventricular thalamic nucleus STR, striatium; VZ, ventricular zone. Scale bar A: 400 µm, B: 200 µm, C: 700, D: 400 µm, E: 200 µm and F: 400 µm.
(TIF)

Figure S2 **Specific immunostaining for anti-RGMa, anti-RGMb and anti-Neogenin antibodies.** COS-7 cells overexpressing RGMa (A, D), RGMb (B, E), GFP-Neogenin (G, J), GFP-DCC (H, K), pcDNA3.1 empty vector (C, F) or pEGFP-N1 (I, L). Cells are counterstained with DAPI in blue. Anti-RGMa and anti-RGMb antibodies specifically stain COS-7 cells overexpressing RGMa (A–C) or RGMb (D–F), respectively. Anti-Neogenin antibody specifically stains COS-7 cells overexpressing GFP-Neogenin and does not stain COS-7 cells overexpressing GFP-DCC or pEGFP-N1 (G–L). Scale bar A-L: 200 µm.
(TIF)

Figure S3 **RGMa-AP binding to E16.5 mouse brain slices.** (A) RGMa-AP binding is detected in cells and neuronal projections in the cortical plate (CP) and intermediate zone (IZ) of the cortex. (B) The fimbria (FIM) of the hippocampus (Hip) and axonal projections in the internal capsule (IC) also bind RGMa-AP. In the hindbrain, the pontine nucleus (PN) and cerebellum (CB), in particular the external granular layer (EGL), are strongly stained for RGMa-AP. Scale bars A–C: 400 µm. CA, cornus ammonis; DG, dentate gyrus; Hb, habenula; STR, striatum; Th, thalamus; VZ ventricular zone.
(TIF)

Table S1 **Sense and antisense primer sequences for** ***RGMa*** **,** ***RGMb*** **,** ***Neo*** **and** ***Unc5A-D in situ*** **hybridization probes.**
(DOCX)

Table S2 **Expression of** ***RGMa*** **,** ***RGMb*** **, Neogenin and** ***Unc5A-D*** **in the primary olfactory system.**
(DOCX)

Table S3 **Expression of** ***RGMa*** **,** ***RGMb*** **,** ***Neogenin*** **and** ***Unc5A-D*** **in the cortex.**
(DOCX)

Table S4 **Expression of** ***RGMa*** **,** ***RGMb*** **,** ***Neogenin*** **and** ***Unc5A-D*** **in the hippocampus and entorhinal cortex.**
(DOCX)

Table S5 **Expression of** ***RGMa*** **,** ***RGMb*** **,** ***Neogenin*** **and** ***Unc5A-D*** **in the habenula, septum and thalamic area.**
(DOCX)

Table S6 **Expression of** ***RGMa*** **,** ***RGMb*** **,** ***Neogenin*** **and** ***Unc5A-D*** **in the cerebellum.**
(DOCX)
