# Supplementary figures and images for: Spatiotemporal Expression of Repulsive Guidance Molecules (RGMs) and Their Receptor Neogenin in the Mouse Brain
Source: PLoS One. 2013 Feb 14;8(2):e55828. doi: 10.1371/journal.pone.0055828 (PMC3573027; doi:10.1371/journal.pone.0055828)

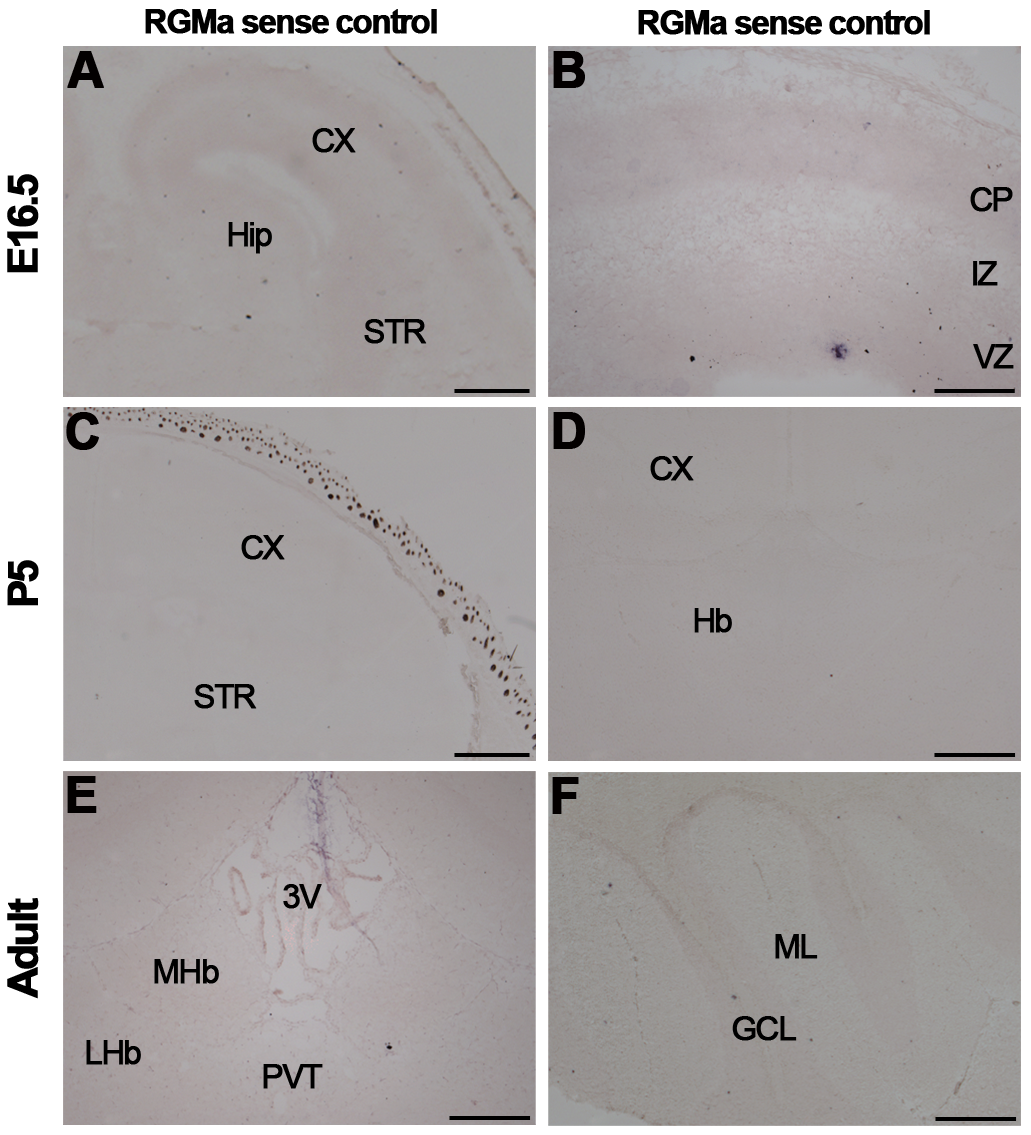

Supplement: Figure S1 — No specific staining for sense probes. In situ hybridization on coronal mouse brain sections at E16.5 (A–B), P5 (C–D) and in adulthood (E–F) using RGMa sense probes. No specific in situ hybridization signals were detected at any of the timepoints or in any of the brain regions examined. Sections hybridized with sense probes for RGMb, Neogenin, and Unc5A-D displayed similar levels of background labeling (not shown). 3V, third ventricle; CP, cortical plate; CX, cortex; GCL, granular cell layer; Hb, habenula; Hip, hippocampus; IZ, intermediate zone; LHb, lateral habenula; MHb, medial habenula; ML, molecular layer; PVT, paraventricular thalamic nucleus STR, striatium; VZ, ventricular zone. Scale bar A: 400 µm, B: 200 µm, C: 700, D: 400 µm, E: 200 µm and F: 400 µm. (TIF) [file pone.0055828.s001.tif]

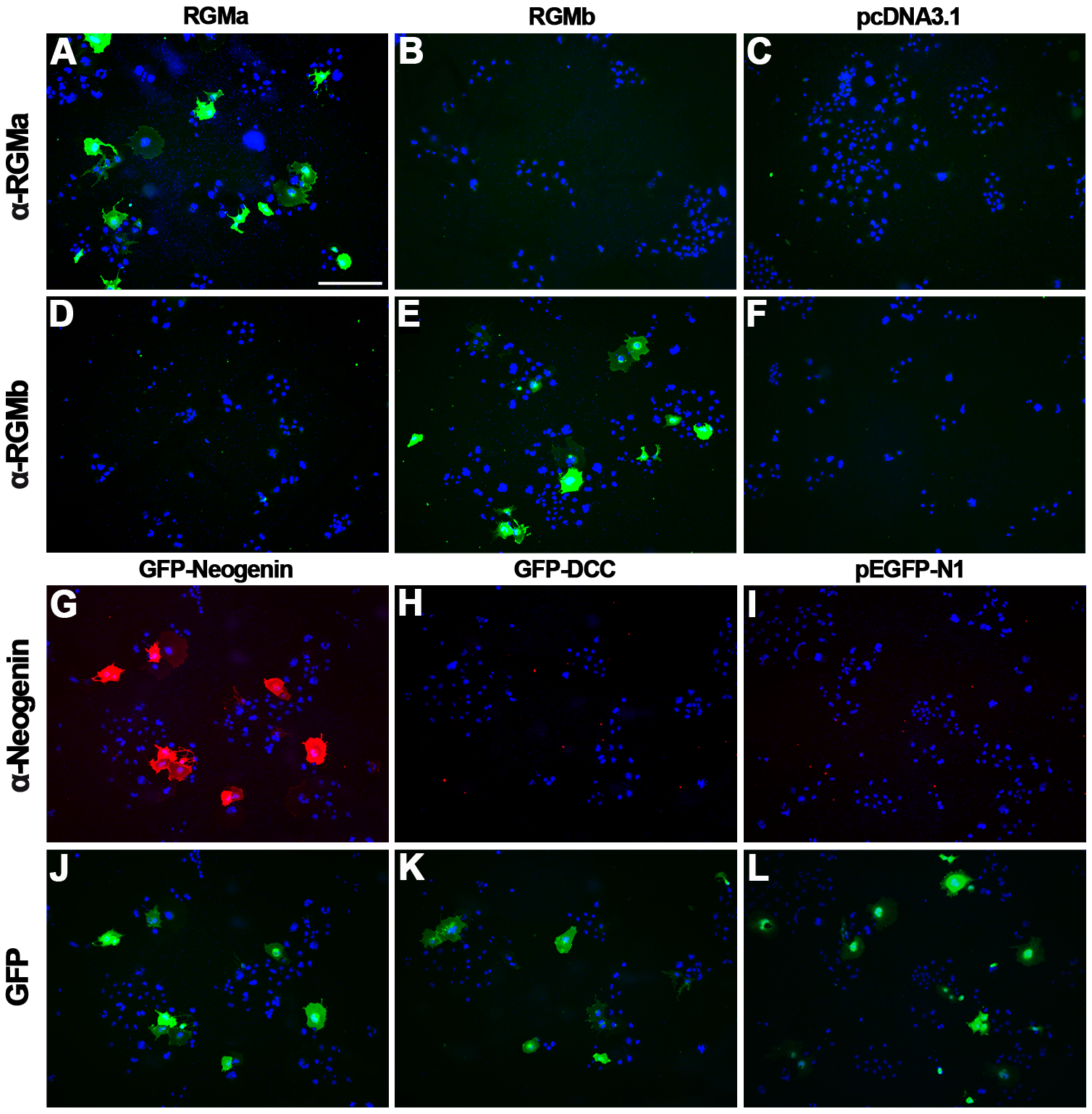

Supplement: Figure S2 — Specific immunostaining for anti-RGMa, anti-RGMb and anti-Neogenin antibodies. COS-7 cells overexpressing RGMa (A, D), RGMb (B, E), GFP-Neogenin (G, J), GFP-DCC (H, K), pcDNA3.1 empty vector (C, F) or pEGFP-N1 (I, L). Cells are counterstained with DAPI in blue. Anti-RGMa and anti-RGMb antibodies specifically stain COS-7 cells overexpressing RGMa (A–C) or RGMb (D–F), respectively. Anti-Neogenin antibody specifically stains COS-7 cells overexpressing GFP-Neogenin and does not stain COS-7 cells overexpressing GFP-DCC or pEGFP-N1 (G–L). Scale bar A-L: 200 µm. (TIF) [file pone.0055828.s002.tif]

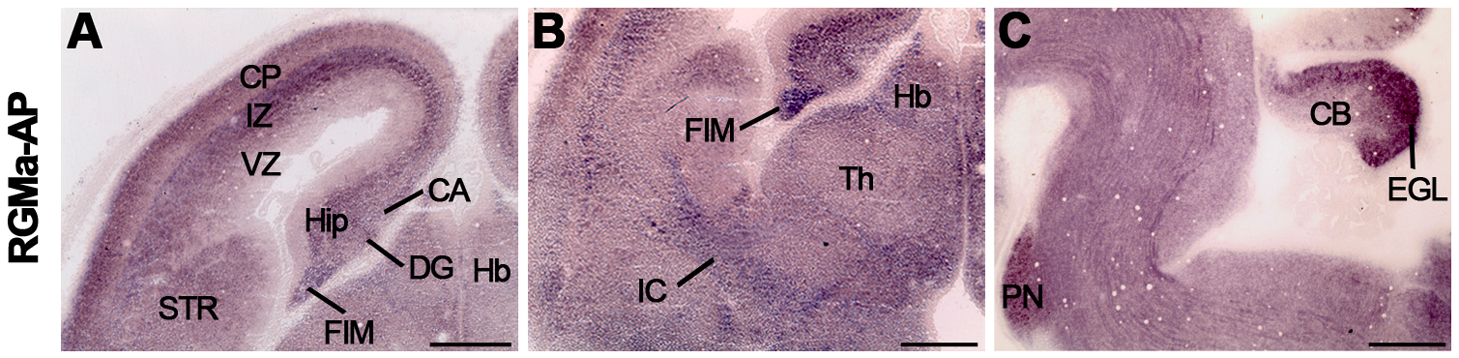

Supplement: Figure S3 — RGMa-AP binding to E16.5 mouse brain slices. (A) RGMa-AP binding is detected in cells and neuronal projections in the cortical plate (CP) and intermediate zone (IZ) of the cortex. (B) The fimbria (FIM) of the hippocampus (Hip) and axonal projections in the internal capsule (IC) also bind RGMa-AP. In the hindbrain, the pontine nucleus (PN) and cerebellum (CB), in particular the external granular layer (EGL), are strongly stained for RGMa-AP. Scale bars A–C: 400 µm. CA, cornus ammonis; DG, dentate gyrus; Hb, habenula; STR, striatum; Th, thalamus; VZ ventricular zone. (TIF) [file pone.0055828.s003.tif]
