# Supplementary material for: Spatiotemporal Expression of Repulsive Guidance Molecules (RGMs) and Their Receptor Neogenin in the Mouse Brain
Source: PLoS One. 2013 Feb 14;8(2):e55828. doi: 10.1371/journal.pone.0055828 (PMC3573027; doi:10.1371/journal.pone.0055828)
Supplement: Table S1 — Sense and antisense primer sequences for RGMa , RGMb , Neo and Unc5A-D in situ hybridization probes. (DOCX) [file pone.0055828.s004.docx]

**Table S1. Sense and antisense primer sequences for *RGMa*, *RGMb*, *Neo* and *Unc5A-D* *in situ* hybridization probes.**

| **Gene** | **Sense primer** | **Antisense primer** | **Size** |
| --- | --- | --- | --- |
| *RGMa* | 5’-TCAGCTGCCCCCAACTACACT-3’ | 5’-TCCTCCACGGCGTTGACTACC-3’ | 455 bp |
| *RGMb* | 5’-CAGCCACGGGGGAGTCAGAG-3’ | 5’-CATCCGGATAGCGAGGGTTAG-3’ | 460 bp |
| *Neo* | 5’-ACACCGTTATCTGGCAATGG-3’ | 5’-TTCAGCAGACAGCCAATCAG-3’ | 501 bp |
| *Unc5A* | 5’-TGAGGTTGCCCCTAGCTG-3’ | 5’-GCTAGAGTTCGCCAGTCG-3’ | 880 bp |
| *Unc5B* | 5’-CGAGTGGGTCAGCCAGAATG-3’ | 5’-CCTCGGCCACAGCGATT-3’ | 546 bp |
| *Unc5C* | 5’-ATTGTGGCTGGGGTATCCTC-3’ | 5’-CAACTGGCTCCTCTTTCTTTCC-3’ | 714 bp |
| *Unc5D* | 5’-AGCGGAGTACCATGGCAAGAATC-3’ | 5’-CTGCCTCCGGAGAAGAAACAGAC-3’ | 1391 bp |
