# Supplementary material for: Spatiotemporal Expression of Repulsive Guidance Molecules (RGMs) and Their Receptor Neogenin in the Mouse Brain
Source: PLoS One. 2013 Feb 14;8(2):e55828. doi: 10.1371/journal.pone.0055828 (PMC3573027; doi:10.1371/journal.pone.0055828)
Supplement: Table S2 — Expression of RGMa , RGMb , Neogenin and Unc5A-D in the primary olfactory system. (DOCX) [file pone.0055828.s005.docx]

**Table S2. Expression of *RGMa*, *RGMb*, *Neogenin* and *Unc5A-D* in the primary olfactory system.**

| **Age** |  | ***RGMa*** | ***RGMb*** | ***Neo*** | ***Unc5A*** | ***Unc5B*** | ***Unc5C*** | ***Unc5D*** |
| --- | --- | --- | --- | --- | --- | --- | --- | --- |
| **E16.5** | Glomerular layer (GL) | + | ++ | +/- | +/- | +/- | +/- | +/- |
|  | External plexiform layer (EPL) | +/- | +/- | +/- | +/- | +/- | +/- | +/- |
|  | Mitral cell layer (MCL) | +++ | +++ | ++ | ++ | +++ | ++ | ++ |
|  | Internal plexiform layer (IPL) | + | +++ | + | + | + | + | + |
|  | Granule cell layer (GR) | + | +++ | + | + | + | ++ | + |
|  | Olfactory ventricular zone (OVZ) | ++ | - | +++ | - | - | - | +++ |
|  | Olfactory epithelium (OE) | ++^a^ | ++^b^ | +^a^ | - | +++ | - | + |
|  | Acessory olfactory bulb (AOB) | ++ | + | ++ | + | ++ | + | + |
|  | Cribriform plate (CRP) | - | - | + | - | - | +++ | - |
| **P5** | Glomerular layer | + | + | + | - | +/- | +/- | +/- |
|  | External plexiform layer | +/- | +/- | +/- | - | - | - | - |
|  | Mitral cell layer | +++ | +++ | + | +/- | + | + | + |
|  | Internal plexiform layer | +/- | +/- | +/- | - | - | - | - |
|  | Granule cell layer | +/- | ++ | ++ | +/- | +/- | +/- | +/- |
|  | Olfactory ventricular zone | + | ++ | + | - | - | + | + |
|  | Olfactory epithelium | + ^a^ | ++ ^b^ | + ^a^ | - | ++ | - | - |
|  | Anterior olfactory nucleus | ++ | ++ | ++ | ++ | - | + | ++ |
|  | Cribriform plate (CRP) | - | - | + | - | - | + | - |
| **Adult** | Glomerular layer | ++ | ++ | ++ | - | + | +/- | +/- |
|  | External plexiform layer | +/- | +/- | +/- | - | - | - | - |
|  | Mitral cell layer | ++ | ++ | + | + | + | + | + |
|  | Internal plexiform layer | +/- | +/- | +/- | - | - | - | - |
|  | Granule cell layer | + | + | + | +/- | +/- | +/- | - |
|  | Olfactory ventricular zone | + | - | + | - | + | + | +/- |
|  | Anterior olfactory nucleus | + | ++ | ++ | ++ | - | ++ | +/- |

^a^ apical expression, ^b^ basal expression.

Legend: - , no expression; +/-, weak expression; + moderate expression; ++, strong expression; +++, very strong expression.
