# Supplementary material for: Spatiotemporal Expression of Repulsive Guidance Molecules (RGMs) and Their Receptor Neogenin in the Mouse Brain
Source: PLoS One. 2013 Feb 14;8(2):e55828. doi: 10.1371/journal.pone.0055828 (PMC3573027; doi:10.1371/journal.pone.0055828)
Supplement: Table S3 — Expression of RGMa , RGMb , Neogenin and Unc5A-D in the cortex. (DOCX) [file pone.0055828.s006.docx]

**Table S3. Expression of *RGMa*, *RGMb*, *Neogenin* and *Unc5A-D* in the cortex.**

| **Age** |  | ***RGMa*** | ***RGMb*** | ***Neo*** | ***Unc5A*** | ***Unc5B*** | ***Unc5C*** | ***Unc5D*** |
| --- | --- | --- | --- | --- | --- | --- | --- | --- |
| **E16.5** | Pia | - | ++ | + | - | + | - | - |
|  | Marginal zone (MZ) | - | + | + | - | - | - | - |
|  | Cortical plate (CP) | +++ | ++^a^ | +++^a^ | ++ | +/- | + | - |
|  | Subplate (SP) | - | + | ++ | + | - | ++ | - |
|  | Intermediate zone (IZ) | - | + | + | +/- | - | - | - |
|  | Subventricular zone (SVZ) | - | ++ | ++ | + | + | +/- | ++ |
|  | Ventricular zone (VZ) | ++ | - | + | + | + | +/- | +/- |
| **P5** | Cortical layer 1 | ++ | - | ++ | +/- | - | + | + |
|  | Cortical layer 2 | ++ | - | ++ | +/- | - | + | + |
|  | Cortical layer 3 | ++ | - | ++ | +/- | - | ++ | + |
|  | Cortical layer 4 | - | - | + | +/- | - | + | + |
|  | Cortical layer 5 | +++ | ++ | +++ | +/- | - | + | - |
|  | Cortical layer 6 | ++ | - | - | +/- | - | - | - |
|  | Ventricular zone | + | - | + | - | - | +/- | - |
| **Adult** | Cortical layer 1 | - | + | +/- | +/- | - | - | + |
|  | Cortical layer 2 | - | + | +/- | +/- | - | - | + |
|  | Cortical layer 3 | - | + | +/- | +/- | - | - | + |
|  | Cortical layer 4 | - | + | +/- | +/- | - | - | + |
|  | Cortical layer 5 | ++ | ++ | +/- | + | - | +/- | - |
|  | Cortical layer 6 | +/- | + | +/- | +/- | - | - | - |
|  | Ventricular zone | + | + | + | - | - | - | - |

^a^ strongest expression in upper part

Legend: - , no expression; +/-, weak expression; + moderate expression; ++, strong expression; +++, very strong expression.
