# Supplementary material for: Spatiotemporal Expression of Repulsive Guidance Molecules (RGMs) and Their Receptor Neogenin in the Mouse Brain
Source: PLoS One. 2013 Feb 14;8(2):e55828. doi: 10.1371/journal.pone.0055828 (PMC3573027; doi:10.1371/journal.pone.0055828)
Supplement: Table S4 — Expression of RGMa , RGMb , Neogenin and Unc5A-D in the hippocampus and entorhinal cortex. (DOCX) [file pone.0055828.s007.docx]

**Table S4. Expression of *RGMa*, *RGMb*, *Neogenin* and *Unc5A-D* in the hippocampus and entorhinal cortex.**

| **Age** |  | ***RGMa*** | ***RGMb*** | ***Neo*** | ***Unc5A*** | ***Unc5B*** | ***Unc5C*** | ***Unc5D*** |
| --- | --- | --- | --- | --- | --- | --- | --- | --- |
| **E16.5** | Hippocampus (Hip) | ++ | + | +++ | ++ | +/- | +/- | + |
|  | Dentate gyrus (DG) | ++ | + | +++ | ++ | + | + | + |
|  | Entorhinal cortex (CEn) | ++ | ++ | +++ | ++ | +/- | - | + |
|  | CA | ++ | ++ | +++ | + | ++ | +/- | - |
|  | Subventricular zone (SVZ) | - | ++ | +/- | + | + | +/- | ++ |
|  | Ventricular zone (VZ) | ++ | - | + | + | + | +/- | +/- |
| **P5** | CA1 | ++ | + | ++ | + | - | +/- | - |
|  | CA2 | + | ++ | ++ | + | - | +/- | - |
|  | CA3 | + | +++ | ++++ | ++ | - | ++ | ++ |
|  | Dentate gyrus | + | + | ++ | + | - | +/- | +/- |
|  | Molecular layer (ML) | + | +/- | + | - | - | +/- | - |
|  | Granular layer (GC) | + | +/- | ++ | + | - | +/- | +/- |
|  | Polymorph layer (PO) | + | + | + | - | - | +/- | + |
|  | Stratum oriens (SO) | +/- | +/- | +/- | - | - | - | +/- |
|  | Stratum radiatum (SR) | +/- | +/- | - | - | - | - | - |
|  | Stratum lacunosum moleculare (SLM) | +/- | + | +/- | - | - | - | - |
|  | Entorhinal cortex (CEn) | ++ | ++ | ++ | +/- | - | - | + |
| **Adult** | CA1 | +++ | ++ | + | ++ | +/- | +/- | +/- |
|  | CA2 | ++ | ++ | + | + | +/- | + | +/- |
|  | CA3 | + | +++ | + | + | +/- | ++ | + |
|  | Dentate gyrus | ++ | ++ | + | ++ | +/- | + | +/- |
|  | Molecular layer | - | - | - | - | - | - | - |
|  | Granular layer | ++ | ++ | + | ++ | +/- | + | +/- |
|  | Polymorph layer | +/- | ++ | + | - | - | +/- | +/- |
|  | Stratum oriens | - | + | - | - | - | - | - |
|  | Stratum radiatum | - | - | - | - | - | - | - |
|  | Stratum lacunosum moleculare | +/- | +/- | - | - | - | - | - |
|  | Entorhinal cortex | +/- | +/- | ++ | ++ | - | - | - |

Legend: - , no expression; +/-, weak expression; + moderate expression; ++, strong expression; +++, very strong expression.
