# Supplementary material for: Spatiotemporal Expression of Repulsive Guidance Molecules (RGMs) and Their Receptor Neogenin in the Mouse Brain
Source: PLoS One. 2013 Feb 14;8(2):e55828. doi: 10.1371/journal.pone.0055828 (PMC3573027; doi:10.1371/journal.pone.0055828)
Supplement: Table S5 — Expression of RGMa , RGMb , Neogenin and Unc5A-D in the habenula, septum and thalamic area. (DOCX) [file pone.0055828.s008.docx]

**Table S5. Expression of *RGMa*, *RGMb*, *Neogenin* and *Unc5A-D* in the habenula, septum and thalamic area.**

| **Age** |  | ***RGMa*** | ***RGMb*** | ***Neo*** | ***Unc5A*** | ***Unc5B*** | ***Unc5C*** | ***Unc5D*** |
| --- | --- | --- | --- | --- | --- | --- | --- | --- |
| **E16.5** | Lateral habenula (LHb) | + | +++ | + | +++ | +/- | + | + |
|  | Medial habenula (MHb) | +++ | +++ | + | ++ | +/- | - | - |
|  | Interpeduncular nucleus (IPN) | ++ | ++ | ++ | ++ | +/- | ++ | ++ |
|  | Substantia nigra (SN) | ++ | + | + | - | - | + | + |
|  | Ventral tegmental area (VTA) | ++ | ++ | + | + | - | + | + |
|  | Septum (S) | + | + | ++ | ++ | + | + | ++ |
|  | Striatum (STR) | + | +++ | + | +/- | + | + | + |
|  | Thalamus (Th) | +++ | ++ | ++ | +++ | - | ++ | - |
|  | Lateral hypothalamus (LH) | + | ++ | ++ | ++ | +/- | ++ | ++ |
| **P5** | Lateral habenula | ++ | +++ | + | +/- | - | - | + |
|  | Medial habenula | +++ | +++ | + | +/- | - | - | - |
|  | Interpeduncular nucleus | + | + | +/- | - | - | - | - |
|  | Substantia nigra | + | +/- | + | - | - | + | + |
|  | Ventral tegmental area | + | +/- | + | - | - | + | + |
|  | Septum | + | +/- | ++ | +/- | +/- | - | + |
|  | Striatum | +/- | +/- | +/- | - | - | - | - |
|  | Thalamus | +++ | + | ++ | +/- | - | + | +/- |
|  | Lateral hypothalamus | + | + | + | - | - | - | +/- |
|  | Paraventricular thalamic nucleus (PVT) | + | ++ | +++ | - | - | + | +/- |
| **Adult** | Lateral habenula | + | +++ | +/- | - | +/- | - | - |
|  | Medial habenula | ++ | ++ | +/- | - | +/- | - | - |
|  | Interpeduncular nucleus | ++ | ++ | + | - | - | - | - |
|  | Substantia nigra | + | + | + | - | - | - | - |
|  | Ventral tegmental area | + | + | + | - | - | - | - |
|  | Septum | + | + | + | + | +/- | - | +/- |
|  | Striatum | - | +/- | - | - | - | - | - |
|  | Thalamus | + | + | + | - | +/- | + | - |
|  | Lateral hypothalamus | +/- | + | +/- | - | - | - | - |
|  | Paraventricular thalamic nucleus | + | + | + | - | + | _­_+ | - |

Legend: - , no expression; +/-, weak expression; + moderate expression; ++, strong expression; +++, very strong expression.
