# Supplementary material for: Spatiotemporal Expression of Repulsive Guidance Molecules (RGMs) and Their Receptor Neogenin in the Mouse Brain
Source: PLoS One. 2013 Feb 14;8(2):e55828. doi: 10.1371/journal.pone.0055828 (PMC3573027; doi:10.1371/journal.pone.0055828)
Supplement: Table S6 — Expression of RGMa , RGMb , Neogenin and Unc5A-D in the cerebellum. (DOCX) [file pone.0055828.s009.docx]

**Table S6. Expression of *RGMa*, *RGMb*, *Neogenin* and *Unc5A-D* in the cerebellum.**

| **Age** |  | ***RGMa*** | ***RGMb*** | ***Neo*** | ***Unc5A*** | ***Unc5B*** | ***Unc5C*** | ***Unc5D*** |
| --- | --- | --- | --- | --- | --- | --- | --- | --- |
| **E16.5** | External granular layer (EGL) | - | + ^a^ | ++ | + | ++ | ++ | - |
|  | Purkinje cell layer (PCL) | + | + | ++ | + | + | ++ | ++ |
|  | Ventricular zone (VZ) | ++ | - | ++ | - | +/- | +++ | - |
|  | Deep cerebellar nuclei (DCN) | ++ | ++ | ++ | ++ | + | +++ | ++ |
| **P5** | External granular layer | - | ++ ^a^ | ++^b^ | - | + ^a^ | ++ | - |
|  | Purkinje cell layer | - | + | ++ | - | + | ++ | + |
|  | Internal granular layer (IGL) | +++ | + | +++ | + | + | ++ | - |
| **Adult** | Molecular layer (ML) | - | - | - | - | - | - | - |
|  | Purkinje cell layer | ++ | ++ | + | + | + | ++ | + |
|  | Granular cell layer (GCL) | + | ++ | ++ | + | + | ++ | - |

^a^ inner EGL layers, ^b^ outer EGL layers

Legend: - , no expression; +/-, weak expression; + moderate expression; ++, strong expression; +++, very strong expression.
